# The role of programmed cell death in chronic obstructive pulmonary disease: from pathogenesis to treatment

**DOI:** 10.3389/fimmu.2026.1715665

**Published:** 2026-03-16

**Authors:** Juandi Xue, Caixia Wang, Hongyan Fan

**Affiliations:** The First People's Hospital of Lanzhou (The Second Clinical Medical College of Gansu University of Chinese Medicine), Lanzhou, Gansu, China

**Keywords:** chronic obstructive pulmonary disease (COPD), ferroptosis, immune homeostasis, immunometabolism, necroptosis, programmed cell death (PCD), pyroptosis

## Abstract

Chronic obstructive pulmonary disease (COPD) is a complex chronic disease characterized by persistent respiratory symptoms and irreversible airflow limitation, and has become a significant global public health issue. Its pathogenesis is highly complex, involving airway inflammation, immune imbalance, oxidative stress, and multiple abnormalities at the cellular and molecular levels. Immunologically, COPD represents a chronic state of “immune homeostasis imbalance” and “immune surveillance failure,” coexisting with persistent activation of innate immunity and dysfunction of adaptive immunity. In recent years, research on programmed cell death (PCD) has gradually gained attention. Especially in the development of COPD, various forms of PCD, including apoptosis, necroptosis, pyroptosis, and ferroptosis, have shown significant biological significance in airway epithelial injury, immune response regulation, and tissue remodeling. This review proposes a core immunological proposition: PCD serves as a key “bridge,” amplifying the innate immune response through mechanisms such as DAMPs release, NLRP3 inflammasome, and immunogenic cell death on one hand, while driving adaptive immune disorders in COPD by affecting antigen presentation, Th1/Th2/Th17 imbalance, T cell exhaustion, and “autoimmune-like” responses on the other hand. This article reviews the roles and molecular mechanisms of various PCDs (apoptosis, necroptosis, ferroptosis, pyroptosis, and copper death) in COPD. It also discusses the associations between different types of PCD, as well as the signaling pathways and regulatory mechanisms of PCD, integrating existing evidence within immunological frameworks such as “immunogenic vs. immunosuppressive cell death,” “defective efferocytosis,” and “Th1/Th2/Th17 imbalance and immunometabolism.” By integrating the latest research findings, it provides a new strategy for targeting PCD in the treatment of COPD. This article aims to provide a deeper immunological understanding of the pathological mechanisms of COPD and to offer new ideas and directions for future therapeutic targets and strategies.

## 1 Introduction

Chronic obstructive pulmonary disease (COPD) is a chronic inflammatory airway disease characterized by persistent airflow limitation as a clinical phenotype, driven by long-term inflammatory responses. It is accompanied by a series of interwoven pathological features, including airway/lung parenchymal cell damage and death, structural remodeling, and immune dysfunction (1, 2). According to data from the World Health Organization, COPD has become one of the leading causes of death globally and is expected to continue to impose a high disease burden in the coming decades (3). The core pathological changes include small airway inflammation and remodeling as well as emphysema, commonly seen in populations with long-term smoking or exposure to pollutants (4, 5). The formation of airflow limitation is closely related to small airway wall thickening, excessive mucus secretion, fibrosis, and destruction of alveolar structures, leading to progressive decline in lung function (6, 7). In addition to structural abnormalities, COPD also manifests as a persistent immune-inflammatory imbalance (including immune cell recruitment and phenotype shift, prolonged elevation of inflammatory mediators, and clearance defects), laying the foundation for chronicity and recurrent acute exacerbations of the disease (8, 9). From an immunological framework, COPD can be viewed as a syndrome of long-term disruption of “immune homeostasis” and “immune surveillance”: innate immunity (such as neutrophils, alveolar macrophages, and NLRP3 inflammasome signaling) is in a state of continuous activation, while adaptive immunity is characterized by T cell exhaustion, imbalance of Th1/Th2/Th17 axis, and impaired regulatory T cell function, both of which shape an inflammatory microenvironment that is difficult to resolve spontaneously (10, 11).

In the aforementioned pathological axis, the abnormal activation and imbalance of cell death programs are important hubs connecting barrier destruction, inflammation amplification, and tissue remodeling (12). Previous studies have suggested that tobacco smoke and pollutant exposure can continuously induce oxidative stress and cellular stress responses, leading to extensive damage and death of airway/alveolar epithelial cells and vascular endothelial cells, disrupting barrier integrity, exacerbating gas exchange impairment, and further promoting inflammation persistence and pathological progression through the release of danger signals (13, 14). From an immunological perspective, programmed cell death (PCD) is not merely “cell death” itself, but continuously drives innate immune amplification through the release of DAMPs, activation of inflammasomes and pattern recognition receptors (PRRs), cytokine/chemokine storms, and immune metabolic reprogramming (15, 16). At the same time, PCD can also alter antigen presentation and co-stimulatory signals, reshape the balance of Th1/Th2/Th17 and Treg, promote CD8^+^ T cell exhaustion and “autoimmune-like” responses, thus forming a critical “bridge” between innate immune activation and adaptive immune dysfunction (17, 18).

However, it is important to emphasize that COPD is not driven by a single “type of PCD.” On the contrary, COPD is more consistent with a dynamic, context-dependent “PCD landscape”: different cell types (airway epithelium, alveolar epithelium, endothelium, and immune cells) initiate different death programs under different stimuli (tobacco smoke, pathogens, particulate matter, persistent oxidative stress, and DAMPs, etc.). The effects of these programs on inflammation and immunity are not consistent and may even “counteract” each other (12, 19). Therefore, treating “PCD” as a homogenized general concept easily obscures the unique and potentially contradictory immunological outcomes of different modes of death in COPD, leading to unclear mechanistic narratives and ambiguous targeting. In immunological terms, the more critical question is not “whether a certain PCD exists in COPD,” but “in which cell lineages, under what immune microenvironment and disease stages, is a specific PCD activated, and through which immunological pathways (such as immunogenic cell death, phagocytic clearance defects, Th cell subset imbalance, and immune metabolic abnormalities) does it influence the disease trajectory” (20).

PCD refers to a cell death process controlled by molecular networks, encompassing multiple pathways with specific key effectors, morphological features, and immunological outcomes. There are also cross-regulatory interactions and state switches among these pathways (21). Overall, apoptosis is generally associated with relative “immunological silence” under effective phagocytic clearance (efferocytosis) conditions, helping to terminate inflammation and maintain tissue homeostasis; in contrast, necroptosis and pyroptosis are often accompanied by membrane rupture/permeabilization and leakage of cellular contents, making it easier to release DAMPs and pro-inflammatory mediators, thereby amplifying local inflammation and promoting tissue destruction and remodeling (22, 23). Meanwhile, one of the core risk factors for COPD—long-term exposure to tobacco smoke—can continuously elevate ROS burden and induce lipid peroxidation, making cell death patterns more likely to shift towards metabolism-oxidative stress-driven death programs, such as ferroptosis; additionally, emerging death modalities related to mitochondrial metabolic stress (such as cuproptosis) are gradually entering the research spotlight (24, 25). Thus, it is evident that “whether PCD is important” is not the key question; what truly determines the pathological trajectory of COPD is which type of PCD is driven in which cell type, under what stimuli or stages, and how the resulting immune-inflammatory consequences shape chronicity and tissue destruction. This definition essentially places PCD at the intersection of immune homeostasis, immune surveillance, and immune metabolic reprogramming, rather than merely viewing it as a “terminal cell fate event.”

Based on this, this review proposes a guiding viewpoint that runs throughout the text: the chronicity and progressive tissue destruction of COPD are crucially linked to the shift in cell death profiles from homeostatic apoptotic clearance to more inflammation-amplifying death programs (such as necroptosis and pyroptosis), which are coupled with metabolism/oxidative stress-driven death modalities (such as ferroptosis and cuproptosis), collectively promoting immune dysfunction, persistent inflammation, and structural tissue damage. At the immunological level, this “PCD landscape drift” is reflected in a series of mutually amplifying abnormal links on the innate immune side, such as DAMPs release, NLRP3 inflammasome and NETs formation, as well as on the adaptive immune side, including imbalance of Th1/Th2/Th17, Treg dysfunction, and T cell exhaustion (26). For example, airway epithelial damage caused by smoking not only directly induces cell death but can also amplify the inflammatory response by activating necroptosis or pyroptosis-related pathways, further exacerbating damage to airway and lung tissue; among them, necroptosis has been shown to be closely related to small airway lesions and the occurrence of emphysema, with its membrane rupture and content release significantly enhancing the local pro-inflammatory environment and worsening airflow limitation (14, 27). Furthermore, oxidative stress is considered a key factor driving the “switching/remodeling” of death programs, and elevated oxidative stress levels in COPD patients are closely related to changes in cell death modes and disease progression (28). To address the therapeutic bottleneck of COPD, an increasing number of studies are beginning to explore intervention strategies targeting key effector molecules from the perspective of “specific death modes—specific cell types—stage-specific microenvironments,” rather than broadly regulating so-called “PCD” (29). These emerging strategies essentially attempt to reshape the dynamic balance between innate and adaptive immunity through fine-tuning PCD, alleviating “cytokine storm”-like inflammation amplification while preserving necessary immune surveillance and anti-infection capabilities (30).

Therefore, this review aims to systematically organize and compare the activation patterns and stimulus-dependent differences of PCD pathways in COPD in various structures and immune cells from a holistic network perspective. It focuses on elucidating how these death programs drive airway damage, persistent inflammation, and structural remodeling through DAMPs release, inflammatory factor network remodeling, phagocytic clearance imbalance, and oxidative-metabolic stress coupling. This article will summarize key evidence and immunological implications of major modes of cell death, including apoptosis, necroptosis, pyroptosis, ferroptosis, and cuproptosis, in the pathogenic mechanisms of COPD, and will further discuss potential intervention points, translational challenges, and future research directions. Through this integrative framework, this review aims to provide a more systematic and in-depth theoretical basis for understanding the immunopathological mechanisms of COPD and its targeted therapeutic strategies.

## 2 Molecular mechanisms of major PCD pathways

**Figure 1:**
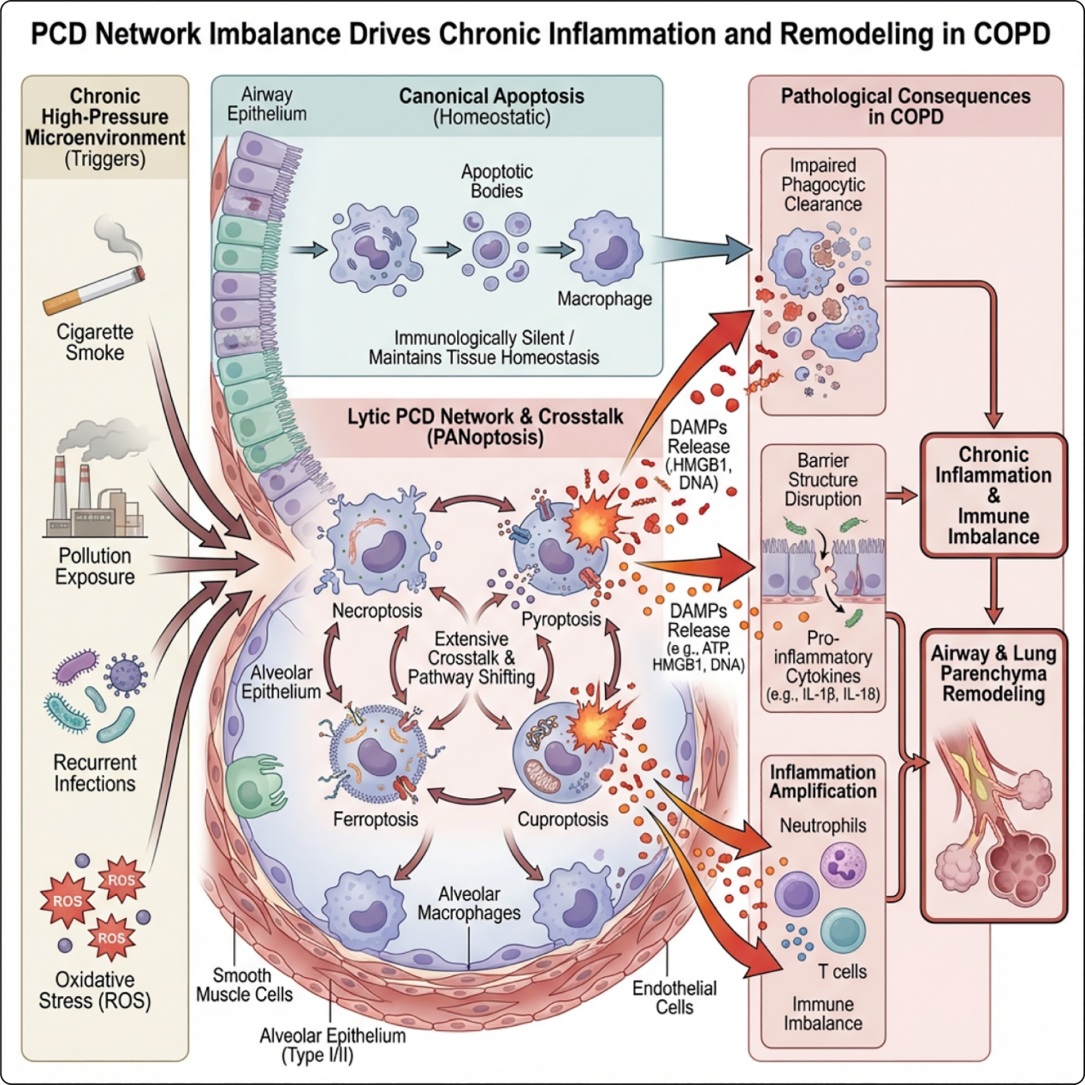
The imbalance of PCD network drives chronic inflammation and remodeling in COPD. Left: “High-pressure” microenvironment factors such as cigarette smoking, pollution exposure, repeated infections, and oxidative stress stimulate the airway and alveolar epithelium. Middle upper: Under homeostatic conditions, apoptotic bodies generated by classical apoptosis can be cleared by macrophages, causing minimal inflammation. Middle lower: In COPD, necroptosis, pyroptosis, ferroptosis, and cuproptosis interact with each other (PANoptosis), leading to the release of a large number of DAMPs and inflammatory mediators. Right: DAMPs induce barrier structure destruction and phagocytic clearance of damaged cells, leading to inflammation amplification. This process is accompanied by immune imbalance involving neutrophils, T cells, and other immune cells, ultimately promoting persistent chronic inflammation and airway/lung parenchyma remodeling.

PCD is a key self-regulatory process for maintaining development and tissue homeostasis in organisms (31). Traditionally, cell death has been primarily classified as apoptosis, characterized by highly controlled cell contraction, nuclear fragmentation, and relatively preserved membrane integrity. Apoptosis usually presents “immunological silence” when effectively phagocytosed, which helps maintain tolerance and homeostasis (32–34). With ongoing research advances, various non-classical PCD modes such as necroptosis, pyroptosis, ferroptosis, and cuproptosis have been identified and further integrated into complex death networks like PANoptosis (35). Compared to classical apoptosis, these novel PCDs are mostly lytic deaths, often accompanied by membrane pore formation or membrane rupture, release of cellular contents and DAMPs, and are more easily coupled directly with inflammation amplification and immune remodeling (36–38) (**Figure 1**). Notably, different death pathways are not isolated from each other, but there is extensive cross-talk and “pathway switching,” which is particularly crucial for understanding COPD characterized by chronic inflammation and tissue remodeling (39, 40).

Before discussing the specific roles of different PCD forms in COPD, we first outline their core molecular mechanisms, which are essential for understanding the specific activation of these death programs in the pulmonary pathological environment. The fundamental physiological function of PCD lies in the clearance of senescent, damaged, or excess cells to maintain tissue homeostasis (41–44). In COPD, long-term smoking, exposure to pollution, recurrent infections, and oxidative stress together create a high-pressure microenvironment, continuously triggering various PCDs in key cell populations such as airway epithelium, alveolar epithelium, smooth muscle, endothelium, and alveolar macrophages. Through mechanisms such as DAMPs/cytokine release, phagocytic clearance barriers, and barrier structure destruction, these processes jointly promote chronic inflammation, immune imbalance, and airway/pulmonary parenchyma remodeling (45, 46). The following will provide a brief overview of the molecular mechanisms of various PCDs and their cellular lineages and functional consequences in the COPD immune microenvironment, to construct an overall picture of “PCD network imbalance” in COPD (**Table 1**).

**Table 1:** Comparative overview of major PCD pathways implicated in COPD.

PCD type	Key initiators/ effectors	Representative markers	Morphological features	Main affected cells	Immunological outcomes	Main evidence levels	Potential intervention targets/strategies
Apoptosis	Exogenous: Fas/FasL, TNF-α/TRAIL–FADD–Caspase-8; Endogenous: Mitochondrial MOMP (Bax/Bak vs Bcl-2/Bcl-XL)–Cyt c–Apaf-1–Caspase-9; Terminal: Caspase-3	Cleaved Caspase-3/8/9; Bax↑/Bcl-2↓; Cyt c release; TUNEL; Annexin V/PI; Mitochondrial membrane potential (JC-1)	Cell shrinkage, nuclear condensation/fragments; Membrane integrity relatively preserved; Formation of apoptotic bodies	Airway/alveolar epithelial cells, alveolar macrophages; Smoking/CSE, air pollution, oxidative stress (ROS), chronic inflammation stimulation	Relatively “immunologically silent” when efferocytosis is intact; Clearance barrier in COPD → accumulation of apoptotic cells → DAMPs (HMGB1, etc.) → PRRs/NF-κB activation, inflammation amplification at moderate to high intensity	*In vitro* (CSE/smoke stimulation of epithelial cells, activation of death receptor axis and mitochondrial pathway); Human (apoptosis coexists with inflammation in BALF; evidence of clearance barrier); Animal: indirect support	Reduce oxidative stress/mitochondrial damage (antioxidation, mitochondrial protection); Restore/Enhance efferocytosis (promote phagocytic clearance); Avoid the risk of infection/bad cell retention caused by broad-spectrum anti-apoptosis.
Necroptosis	RIPK1–RIPK3–MLKL axis; Necrosome formation; p-MLKL oligomerization and insertion into the cell membrane causing lysis	p-RIPK3, p-MLKL; MLKL oligomerization/membrane localization; PI uptake↑; LDH release↑	Cell swelling, membrane rupture; Leakage of cell contents (necrosis-like)	Airway smooth muscle cells, endothelial cells, etc.; Virus/infection-related acute exacerbation; Oxidative stress (ROS), CSE induction	Highly inflammatory: membrane rupture → leakage of DAMPs (HMGB1, etc.) → recruitment of neutrophils and amplification of inflammation (high intensity)	*In vitro* (CSE induces ROS↑ and p-RIPK3/p-MLKL↑); Animal (inhibition of pathway reduces inflammation/remodeling/emphysema); Human: upregulation of expression in acute exacerbation/infection context	RIPK1 inhibitor RIPK3/MLKL inhibition Combined antioxidant/anti-infection strategy; Applicable to the window of high inflammatory load or acute exacerbation (safety stratified assessment is required).
Pyroptosis	Inflammasome (NLRP3, etc.)–ASC–Caspase-1; Non-classical: Caspase-4/5/11; GSDMD cleavage forms membrane pores; IL-1β/IL-18 mature release	NLRP3, ASC speck; cleaved Caspase-1; GSDMD-N; IL-1β/IL-18; PI uptake/LDH↑	Cell swelling, membrane pore formation followed by rupture; Inflammatory necrosis-like	Airway epithelial cells, macrophages/neutrophils, etc.; Smoking/CSE, particulate matter, infection (acute exacerbation)	Highly inflammatory: GSDMD pore formation → leakage of IL-1β/IL-18 and DAMPs, high intensity inflammation; Can drive local to systemic inflammation	*In vitro*/Animal: NLRP3 activation and pyroptosis readout enhanced; Human: clues of acute exacerbation and enhanced inflammasome signaling	NLRP3 inhibition Caspase-1 inhibition Block the IL-1 signal; GSDMD inhibition Emphasize the stratification of infection risk and phenotype (especially acute exacerbation/high inflammatory load).
Ferroptosis	Iron overload↑ (Fe2+); Lipid peroxidation chain reaction; GSH depletion and GPX4 inactivation; System Xc− (SLC7A11) involvement	GPX4↓, GSH↓; Lipid-ROS (C11-BODIPY); MDA/4-HNE; ACSL4↑; Iron levels/TFRC/FERROPORTIN; Mitochondrial morphology changes (electron microscopy)	Mitochondrial shrinkage, reduced/disappeared cristae; Increased membrane density; Not primarily caspase activation-dependent	Alveolar/airway epithelial cells, etc.; Smoking/oxidative stress; Iron metabolism disorders (iron deposition)	Moderate to high intensity: lipid peroxidation and oxidative stress promote inflammation; Can alter immune cell function and metabolic microenvironment, promoting chronic inflammation	*In vitro*/Animal: Inhibition of ferroptosis can reduce inflammation/injury clues; Human: Abnormal iron metabolism and related markers association.	Iron chelation Antioxidant/removal of lipid peroxidation; Strengthen the GPX4-GSH defense line (such as supplementing precursors/activating related pathways); Promote translational validation through “mechanism-biomarker-phenotypic stratification”.
Cuproptosis	Accumulation of free copper ions; Binding to mitochondrial TCA acetylated proteins → protein aggregation; Downregulation of Fe-S proteins; Protein toxicity stress and energy metabolism disorders	Copper levels (tissue/serum); FDX1, DLST, LIPT1, LIAS, etc.; Acetylated protein aggregation (proteomics/immunoblotting); Mitochondrial respiration and TCA functional indicators	Morphological features are still being refined: centered on mitochondrial metabolic stress and protein aggregation; Traditional apoptosis/necroptosis morphology is not the main criterion.	Copper elevation in smoking-related lung tissue; May affect the metabolism of alveolar macrophages and other immune cells; Specific triggering stimuli and cell type localization still need clarification.	Currently mostly correlational clues: associated with immune infiltration/inflammation degree, potential pro-inflammatory (intensity uncertain)	Human: Copper content associated with gene expression; *In vitro*/Animal: Insufficient specific causal evidence for COPD (hypothesis stage)	Targeted copper homeostasis (chelation/transport regulation) is associated with key metabolic nodes (TCA/acylation); Priority completion: Cell type localization, trigger stimulation, core markers, and causal verification of intervention before evaluating transformation.

### 2.1 Apoptosis

Cell apoptosis is a genetically regulated autonomous cell death process, mainly mediated by the extrinsic death receptor pathway and the intrinsic mitochondrial pathway (47). The BCL-2 family (such as pro-apoptotic Bax, Bak, and anti-apoptotic Bcl-2, Bcl-XL) plays a “gatekeeping” role by regulating mitochondrial outer membrane permeability (MOMP), determining cell fate (48). In the extrinsic pathway, activation of death receptors such as Fas/FasL, TNF-α/TNFR1, and TRAIL/DR5 can trigger Caspase-8 activation. In Type I cells, Caspase-8 directly cleaves Caspase-3 to initiate the execution cascade; while in Type II cells, Caspase-8 first mediates mitochondrial amplification through Bid→tBid, promoting the release of cytochrome c and activating the Apaf-1–Caspase-9–Caspase-3 axis, ultimately completing apoptosis (49, 50). Activated Caspase-3 can also cleave Caspase-8 in reverse, forming an amplification loop that consolidates the death signal, thereby resulting in a complete execution network coupled between the “extrinsic-mitochondrial” pathways (51, 52). In the COPD-related immune context, apoptosis mainly occurs in airway/alveolar epithelial cells and alveolar macrophages, which are innate immune cells, and has a bidirectional regulatory effect on the cytokine/chemokine profile, tolerance-inflammation balance, and epithelial barrier structure (53, 54).

In COPD, substantial evidence supports a significant imbalance of apoptosis in airway and alveolar epithelium as well as alveolar macrophages. The levels of TUNEL-positive cells, activated Caspase-3/9, and the Bax/Bcl-2 ratio are significantly elevated in the airway epithelium and alveolar epithelial cells of COPD patients (55, 56). *In vitro* models of human bronchial epithelial cells and alveolar epithelial cells treated with cigarette smoke extract (CSE) show upregulation of Fas, FasL, TNF-α, and TRAIL expression, accompanied by the activation of initiator Caspase-8, executioner Caspase-3 cleavage, and loss of mitochondrial membrane potential, suggesting a coupling of the typical death receptor pathway with the intrinsic mitochondrial pathway in an “extrinsic-mitochondrial amplification” manner (57). Within the classical Type I/Type II apoptosis framework, activated Caspase-8 in some epithelial cells can directly cleave Caspase-3 to induce apoptosis (Type I), while in more airway/alveolar epithelial cells considered Type II, Caspase-8 also drives mitochondrial release of cytochrome c by cleaving Bid to generate tBid, amplifying the Apaf-1–Caspase-9–Caspase-3 cascade. Simultaneously, activated Caspase-3 can cleave Caspase-8 in reverse to form the fully activated p18 fragment, creating a positive feedback loop, thereby consolidating and exacerbating the apoptotic phenotype of epithelial cells under continuous CSE exposure (58). Smoke-exposed animal models also show similar changes. In terms of immune cells, the proportion of apoptotic alveolar macrophages in the bronchoalveolar lavage fluid (BALF) of COPD patients increases, accompanied by sustained activation of NF-κB and high expression of inflammatory factors such as IL-8 and TNF-α. In contrast, the clearance of apoptotic cells (efferocytosis) is significantly impaired, forming a mismatch of “increased apoptotic cell load - decreased phagocytic capacity” (59).

Under physiological conditions, the “immunologically silent” apoptosis occurring in airway and alveolar epithelium can induce macrophages to produce anti-inflammatory factors such as TGF-β and IL-10 through timely efferocytosis, thereby maintaining local tolerance and promoting inflammation resolution. However, in the high-pressure microenvironment of COPD, on one hand, excessive epithelial cell apoptosis leads to thinning of the epithelial barrier, damage to tight junctions, and widening of barrier gaps, weakening mucosal defense and promoting the penetration of pathogens and particulates (60). On the other hand, efferocytosis dysfunction causes a large number of apoptotic cells to remain and undergo secondary necrosis, releasing DAMPs such as HMGB1. These DAMPs are recognized by PRRs (such as TLR4) on the surface of alveolar macrophages and neutrophils, activating the NF-κB pathway, driving the upregulation of pro-inflammatory cytokines and chemokines such as IL-1β, IL-6, IL-8/CXCL8, and TNF-α, enhancing the recruitment of neutrophils and monocytes, thus transforming the originally “relatively tolerant” apoptosis into a powerful pro-inflammatory amplifier (61).

**Figure 2:**
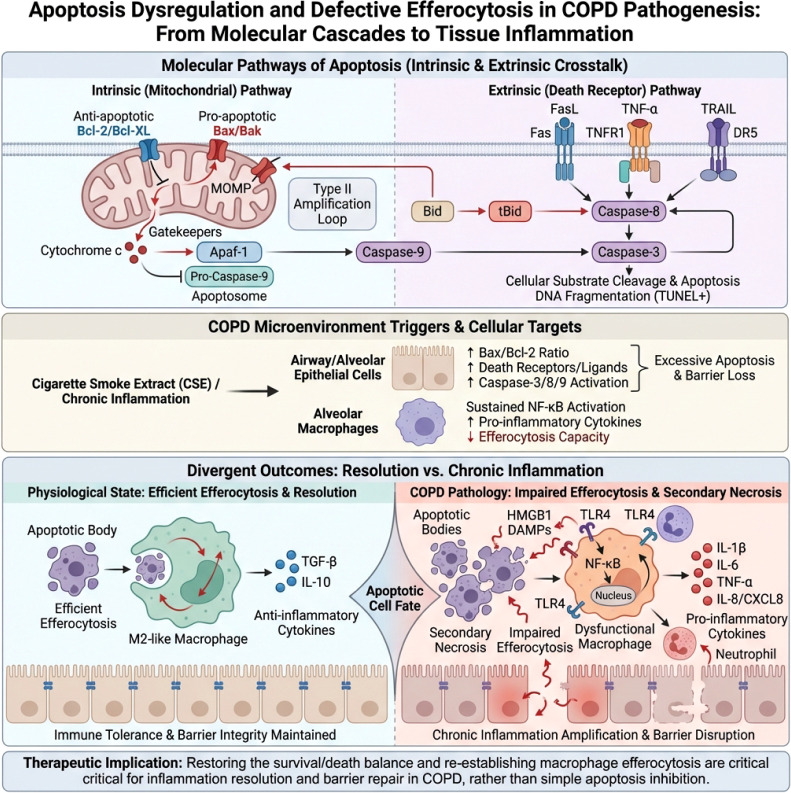
Schematic of the role of apoptosis imbalance and efferocytosis defects in the pathogenesis of COPD. Top: The intrinsic (mitochondrial) and extrinsic (death receptor) pathways jointly drive apoptosis through Bax/Bak-mediated MOMP, the cytochrome c–Apaf-1–Caspase-9 cascade, and the Fas/TNF-α/TRAIL-mediated Caspase-8/-3 activation. Middle: Smoking extracts and chronic inflammation affect the airway and alveolar epithelium as well as macrophages, upregulating the Bax/Bcl-2 ratio and death receptor, Caspase activity, and continuously activating NF-κB, leading to excessive apoptosis and barrier damage. Bottom: Under physiological conditions, apoptotic bodies are efficiently cleared by M2-like macrophages, releasing TGF-β and IL-10, maintaining immune tolerance and epithelial integrity; whereas in COPD, efferocytosis is impaired, leading to many apoptotic cells undergoing secondary necrosis, releasing DAMPs such as HMGB1 and inflammatory cytokines, amplifying chronic inflammation and exacerbating epithelial barrier damage via the TLR4/NF-κB pathway. This figure suggests that restoring the balance of apoptosis and clearance is more critical than simply inhibiting apoptosis.

Therefore, in terms of immune connections, apoptosis in COPD supports TGF-β/IL-10 mediated immune tolerance against a background of efficient efferocytosis. Conversely, it drives a pro-inflammatory cytokine profile characterized by IL-1β/IL-6/CXCL8 during the clearance of damaged tissue and epithelial barrier destruction, promoting the continuation of inflammation by weakening epithelial integrity (**Figure 2**). Based on the above understanding, simply “inhibiting apoptosis” is not an ideal strategy; it is more critical to restore an appropriate survival-to-death balance between structural cells and immune cells, and to rebuild the efferocytosis capacity of macrophages, allowing apoptosis to return to a trajectory favorable for inflammation resolution and barrier repair.

### 2.2 Necroptosis

Necroptosis is a form of programmed necrosis mediated by receptor-interacting protein kinase 1 (RIPK1) and RIPK3 through receptor interactions. Its core feature is the phosphorylation and oligomerization of mixed lineage kinase domain-like protein (MLKL), which inserts into the cell membrane, leading to increased membrane permeability and massive leakage of cellular contents. Therefore, it is often classified as a typical lytic programmed cell death (lytic PCD) (62, 63). In situations where TNFR, pattern recognition receptors (PRRs), or certain viral sensors are activated, and Caspase-8 activity is restricted or inhibited, the RIPK1–RIPK3 complex forms a necrosome, which subsequently activates MLKL and triggers necroptosis (64–66). Oxidative stress and mitochondrial ROS can form a positive feedback loop with this pathway, amplifying local cell death signals and simultaneously enhancing the activation of downstream neuroendocrine and inflammatory pathways (67). In COPD, necroptosis primarily occurs in airway epithelium, airway smooth muscle, pulmonary microvascular endothelium, and immune cells such as monocytes/macrophages and dendritic cells, promoting inflammatory amplification through marked alterations in the profiles of DAMPs and cytokines/chemokines, and indirectly damaging the airway and vascular barrier (68, 69).

In COPD-related studies, necroptosis has been confirmed to be activated in various pulmonary structural cells, particularly closely associated with acute exacerbations of COPD (AECOPD) (70). In patients with virus infection-related acute exacerbations, the expression of RIPK3 and p-MLKL in airway smooth muscle cells and pulmonary microvascular endothelial cells is significantly elevated. These cells present necrotic morphological features, suggesting that necroptosis is involved in airway hyperreactivity and microvascular injury (69). In *in vitro* models, airway epithelial cells treated with CSE and animal models with long-term smoke exposure show a significant increase in intracellular ROS load, accompanied by elevated levels of RIPK3/MLKL phosphorylation; the application of RIPK1/3 or MLKL inhibitors can alleviate small airway lesions and the progression of emphysema, thereby more clearly incorporating necroptosis into the structural injury spectrum of COPD (71–73).

Since necroptosis is essentially a lytic form of cell death, its immune and inflammatory consequences are highly pro-inflammatory. The rupture of the cell membrane releases a substantial amount of DAMPs, including HMGB1, ATP, DNA fragments, and inflammatory mediators such as IL-1α and CXCL1, which strongly activate neutrophil chemotaxis and NET formation, driving neutrophil-dominated inflammatory infiltration (74). It is important to clarify that neutrophil necrosis (NETosis) is not classical PCD, but rather a neutrophil-specific mode of death, highly dependent on the amplification of ROS and DAMPs produced from necroptosis, ferroptosis, and other pathways. In acute exacerbations of COPD, it resembles an “end-stage effector driven by other PCDs,” playing a key role in microvascular and epithelial barrier damage (75, 76).

At the structural cell level, necroptosis in endothelial cells can disrupt the microvascular barrier, increase permeability and cause leakage, alter local shear forces and adhesion molecule expression, thereby promoting the migration of monocytes and lymphocytes into lung tissue, further amplifying inflammation (77, 78). At the immune cell level, when monocytes/macrophages and dendritic cells shift from “relatively silent” apoptosis to necroptosis upon stimulation by pathogen-associated molecular patterns (PAMPs), it is often accompanied by the release of a large amount of cytokines and chemokines such as IL-6, TNF-α, and CCL2, shaping a “high inflammation, low resolution” immune phenotype, weakening immune tolerance and inflammatory resolution pathways (79). Overall, necroptosis tends to push the immune microenvironment of COPD from relative homeostasis towards chronic inflammation and a tendency for acute exacerbations, especially exhibiting an “amplifier” effect during disease stages driven by viral or strong oxidative stress (19).

**Figure 3:**
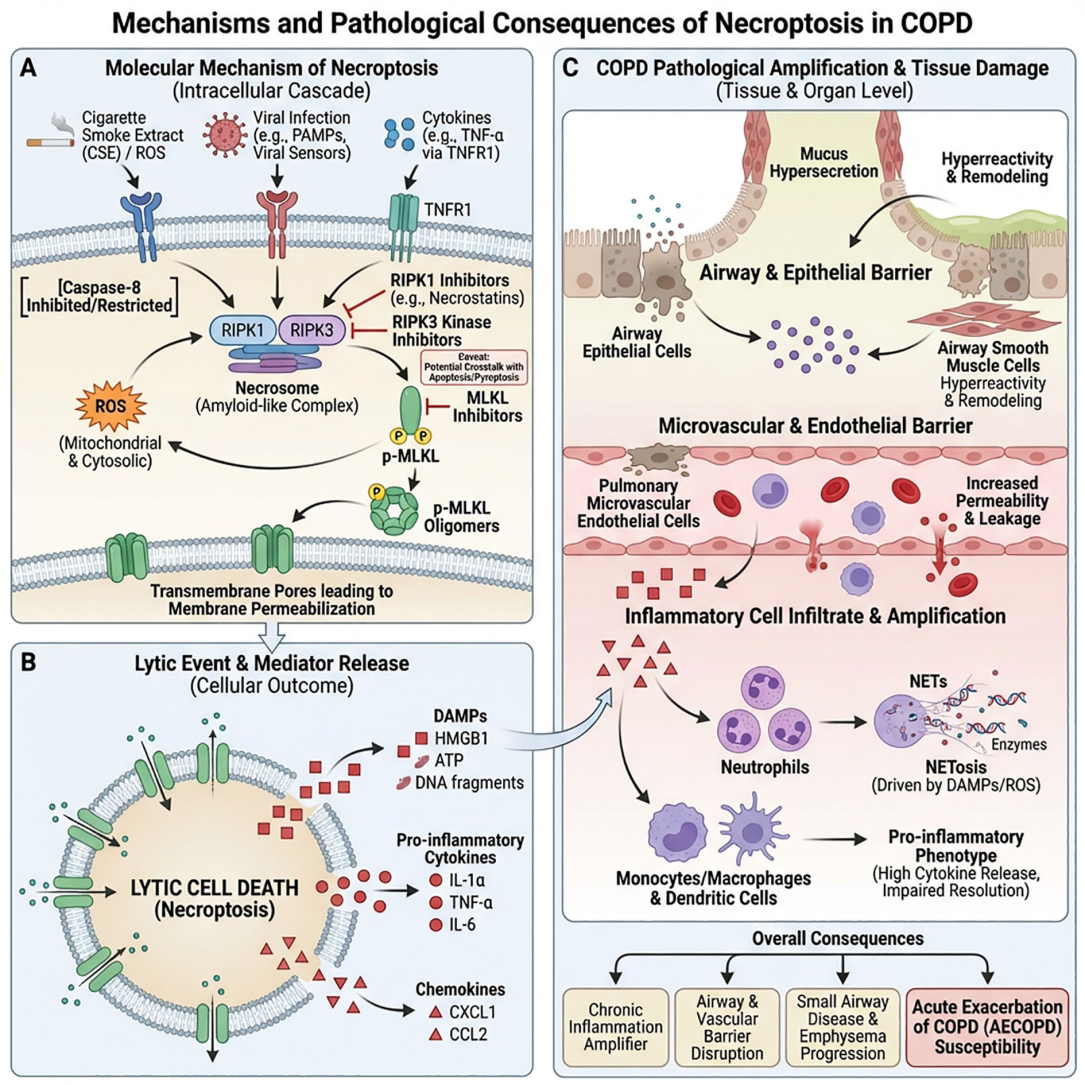
Mechanisms and pathological consequences of programmed necrosis (necroptosis) in COPD. **(A)** Upon stimulation by cigarette extract, reactive oxygen species (ROS), viral infection, and cytokines such as TNF-α, TNFR1 activates the RIPK1–RIPK3 complex and forms a necrosome when Caspase-8 is inhibited. This complex phosphorylates MLKL and forms pores in the cell membrane, accompanied by elevated mitochondrial and cytoplasmic ROS, leading to necrotic-like lytic cell death. **(B)** Lytic cells release DAMPs such as HMGB1 and DNA fragments, as well as pro-inflammatory cytokines like TNF-α and IL-6, and chemokines such as CXCL1 and CCL2, which act as potent inflammatory amplification signals. **(C)** These mediators lead to damage to the airway epithelium and microvascular endothelial barrier, increased permeability, and massive infiltration of neutrophils and monocytes/macrophages forming NETs, inducing excessive mucus secretion, airway hyperreactivity, and structural remodeling of lung parenchyma, overall promoting sustained chronic inflammation, destruction of airways and small airways, and increased susceptibility to acute exacerbations.

In animal experiments, RIPK1 inhibitors, including necrostatins, and small molecules or genetic interventions targeting RIPK3/MLKL can significantly reduce inflammatory cell infiltration and structural damage in lung tissue. This suggests that necroptosis is a druggable regulatory node (80). However, current related interventions remain at the preclinical stage, and caution must be exercised regarding their network effects due to crosstalk with apoptosis, pyroptosis, and other PCD modes—excessive inhibition of necroptosis may lead to a shift of death pathways towards pyroptosis or other lytic PCDs, resulting in unexpected immunological consequences (81, 82). A more prudent strategy involves limiting necroptosis inhibition to the early phase of AECOPD, primarily driven by viral infection or severe oxidative stress, with short-term combined anti-infection and anti-inflammatory treatment to alleviate inflammation and structural damage while avoiding excessive suppression of long-term immune surveillance and host defense (83, 84) (**Figure 3**).

### 2.3 Pyroptosis

Pyroptosis is a form of programmed cell death that is Caspase-1 dependent (or mediated by the non-classical pathway of Caspase-4/5/11) and is highly pro-inflammatory. Its typical morphological features include cell swelling, membrane perforation, and the massive release of IL-1β and IL-18 (85–87). At the molecular level, the NLRP3 inflammasome is the most studied pyroptosis platform in COPD-related research, composed of NLRP3, ASC, and Pro-Caspase-1; this complex upregulates the expression of NLRP3 and Pro-IL-1β/IL-18 in response to the “priming signal” mediated by NF-κB, while it assembles and matures after receiving “activation signals” such as ion flux changes (e.g., K^+^ efflux), ROS, and mitochondrial DNA (mtDNA) (88). After inflammasome activation, Caspase-1 and Caspase-4/5/11 are activated, which promote the cleavage and maturation of Pro-IL-1β and Pro-IL-18, and cleavage of Gasdermin D (GSDMD), resulting in the GSDMD-N terminal fragment inserting into the cell membrane and forming pores, leading to the leakage of cellular contents and lytic cell death (89, 90). In the immune microenvironment of COPD, pyroptosis mainly occurs in myeloid immune cells such as airway epithelial cells, alveolar macrophages, and monocytes, acting as a primary mode of cell death that drives the IL-1 axis and neutrophilic inflammation, significantly impacting airway barrier disruption and loss of immune tolerance (91).

In terms of COPD-related evidence, the expression of NLRP3, ASC, Caspase-1, and GSDMD is generally upregulated in bronchial mucosal biopsies and bronchoalveolar lavage fluid (BALF) from smokers and COPD patients, accompanied by elevated levels of IL-1β and IL-18 (92–94). *In vitro* experiments show that both CSE and particulate matter exposure can induce NLRP3-dependent pyroptosis in airway epithelial cells, alveolar macrophages, and monocytes, characterized by Caspase-1/GSDMD activation and massive release of IL-1β; inhibiting NLRP3 or Caspase-1 significantly reduces membrane perforation and IL-1β release (95). In animal smoke exposure models, NLRP3 or GSDMD gene knockout mice exhibit reduced pulmonary inflammatory infiltration, decreased airway remodeling, and structural damage, further supporting the pathogenic role of pyroptosis in COPD progression at the *in vivo* level (96, 97).

From the perspective of immunity and inflammation, pyroptosis significantly reshapes the immune microenvironment of COPD through the IL-1β/IL-18–CXCL1/2 axis (98). When airway epithelial cells undergo pyroptosis, the epithelial barrier is directly disrupted, tight junctions are damaged, and the basement membrane is exposed, making it easier for pathogens to adhere and invade. When alveolar macrophages and monocytes undergo pyroptosis, a large amount of IL-1β, IL-18, and CXCL1/2 is released, strongly driving neutrophils to aggregate in the airways and alveoli, thereby forming a neutrophil-dominant inflammatory infiltration pattern. This pattern is often accompanied by upregulation of TNF-α, IL-6, and CCL2/5, promoting Th17 responses, weakening Treg-mediated immune tolerance, and shifting the overall immune balance towards a pro-inflammatory phenotype (99, 100). Unlike apoptosis, which is relatively “silent” under efficient clearance, pyroptosis is almost always accompanied by a significant cytokine storm, especially amplifying the host’s excessive response to pathogens and environmental stimuli in infection-related AECOPD, accelerating tissue damage and lung function deterioration (101).

**Figure 4:**
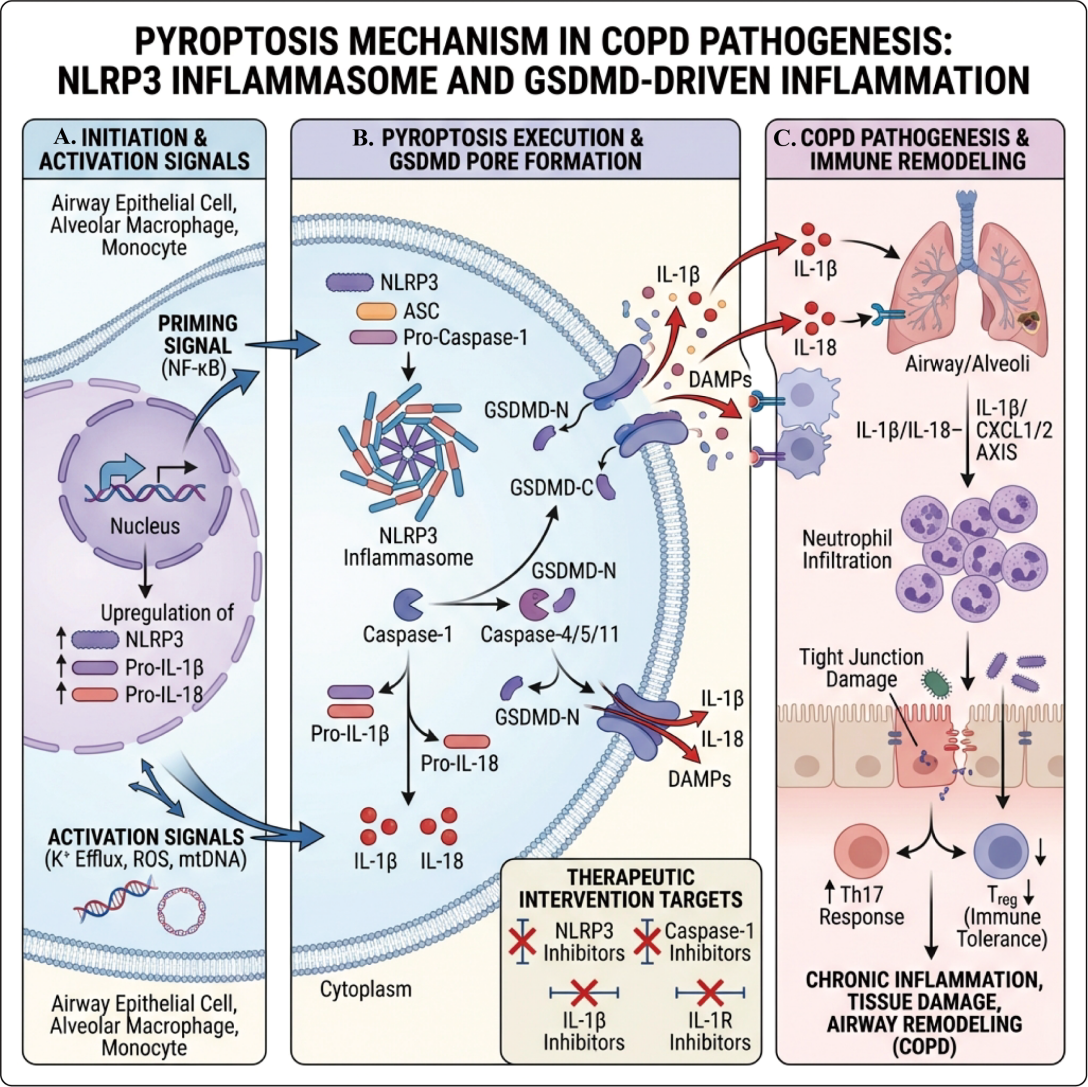
Mechanism of pyroptosis in COPD: NLRP3 inflammasome and GSDMD-mediated inflammation. **(A)** Cigarette smoke, ROS, mtDNA, and other stimuli trigger airway epithelial cells, alveolar macrophages, and monocytes, leading to “priming” mediated by NF-κB, upregulating NLRP3, pro-IL-1β, and pro-IL-18, and activation triggered by signals such as K^+ efflux. **(B)** NLRP3 recruits and activates Caspase-1 (or Caspase-4/5/11), cleaving GSDMD to form GSDMD-N pores, while pro-IL-1β/pro-IL-18 is cleaved into mature IL-1β and IL-18, which are substantially released through membrane pores and cell lysis, accompanied by DAMPs expulsion. **(C)** IL-1β/IL-18 and DAMPs induce neutrophil infiltration, upregulating CXCL1/IL-8 and disrupting tight junctions; they form an immune imbalance characterized by enhanced Th17 responses and impaired Treg function, which drives chronic airway/alveolar inflammation, tissue damage, and airway remodeling. Potential intervention targets are shown below, including NLRP3, Caspase-1, GSDMD, and IL-1/IL-18 signaling inhibitors.

Therapeutically, the NLRP3 inflammasome and its downstream IL-1 axis are considered key targets for intervening in pyroptosis. Various NLRP3 inhibitors, Caspase-1 inhibitors, and blocking strategies targeting the IL-1 axis (such as IL-1β or IL-1R inhibitors) have been shown to significantly reduce airway inflammation and structural damage in COPD animal models, providing strong conceptual validation for clinical translation (102). Future trial designs need to find a balance between “inhibiting excessive inflammation” and “preserving anti-infection defenses,” avoiding a simplistic application of “pan IL-1 inhibition” to all COPD patients, and instead prioritizing phased interventions for populations with high acute exacerbation frequency or high IL-1 phenotype (103) (**Figure 4**).

### 2.4 Ferroptosis

Ferroptosis is a form of programmed cell death that depends on iron ions and lipid peroxidation. Its core mechanism includes three interlinked processes: first, intracellular free iron (Fe²^+^) generates high levels of ROS through the Fenton reaction (104). Second, polyunsaturated fatty acids (PUFA) accumulate in the cell membrane and are oxidized into lipid peroxides under the action of lipoxygenases and other enzyme systems (105). Third, the depletion of glutathione (GSH) and the impairment of glutathione peroxidase 4 (GPX4) function prevent these lipid peroxide products from being timely reduced and cleared, ultimately leading to irreversible damage to membrane structures and cell death (106). In this process, the uptake and transport of iron (including transferrin receptor 1 (TFR1) and divalent metal transporter 1 (DMT1), storage (ferritin), and output (FPN1), as well as the system Xc^−^–GSH–GPX4 axis, together constitute key regulatory nodes of ferroptosis, determining the cell’s susceptibility to iron load and oxidative stress (107, 108). In the COPD-related immune microenvironment, ferroptosis mainly occurs in structural cells such as airway and alveolar epithelium, endothelium, and fibroblasts, and can also affect alveolar macrophages and neutrophils, impacting gas exchange and barrier integrity through structural damage, as well as promoting the maintenance of a chronic pro-inflammatory phenotype by altering cytokine profiles and macrophage polarization (109, 110).

Regarding COPD-related evidence, multiple studies suggest that increased pulmonary iron load is closely related to an increased tendency for ferroptosis. The iron content and ferritin expression in the lung tissue and BALF of COPD patients are generally elevated and positively correlated with smoking load, the degree of decline in lung function, and the severity of emphysema (111–114). In *in vitro* models, airway epithelial cells and alveolar epithelial cells treated with CSE exhibit upregulation of TFR1, redistribution of ferritin, decreased GSH levels, and reduced GPX4 expression, which parallel the accumulation of lipid ROS and increased cell death (45, 115). In animal smoke exposure models, the use of iron chelators or interventions that enhance GPX4 activity can alleviate lung structural damage and inflammatory cell infiltration, functionally supporting the causal role of ferroptosis in COPD structural damage and inflammation amplification (109, 116). At the level of immune cells, abnormal iron load can increase the susceptibility of alveolar macrophages and neutrophils to ferroptosis or iron-related metabolic reprogramming, altering their phagocytosis, bactericidal activity, and inflammatory mediator release profiles, further affecting local immune defense and inflammatory dynamics (117, 118).

From an immune/inflammatory perspective, ferroptosis simultaneously affects structural cells and some immune cells. Epithelial cell ferroptosis can lead to thinning of the alveolar wall, destruction of septa, and reduced effective gas exchange area, which is an important component of structural damage in emphysema (119); during this process, a large amount of oxidized lipids, mtDNA, and DAMPs such as HMGB1 are released, activating alveolar macrophages and dendritic cells via TLRs/NLRs, driving the upregulation of IL-6, TNF-α, CXCL8, and promoting sustained recruitment of neutrophils, forming a positive feedback loop of “iron load ↑– lipid peroxidation ↑– inflammation ↑” (120). Chronic iron load also promotes macrophage polarization towards the M1 type, inhibiting the M2/resolving phenotype, weakening the fine-tuning ability of tissue repair and fibrosis, and causing the COPD immune microenvironment to remain in a low but persistent pro-inflammatory state (121).

**Figure 5:**
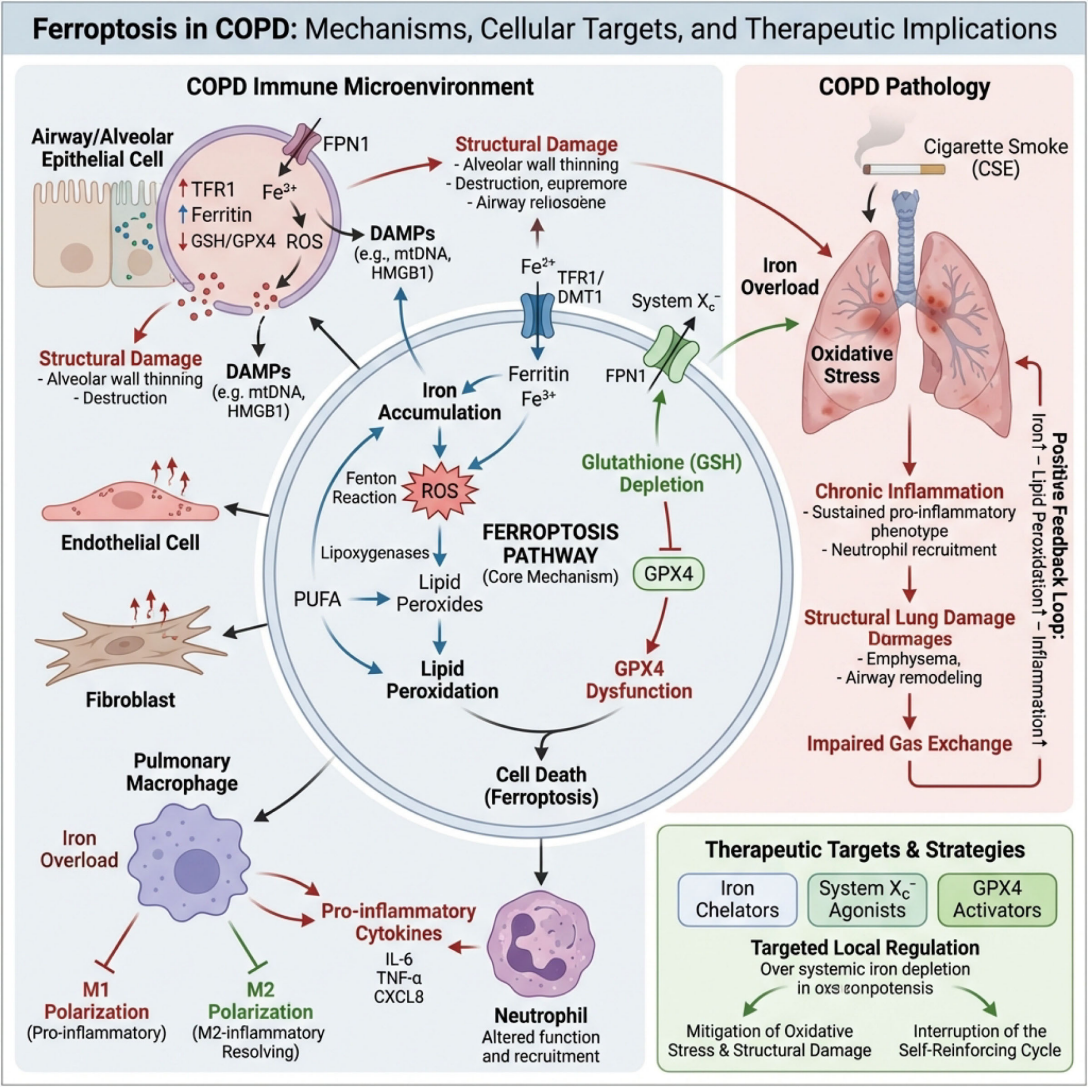
Ferroptosis in COPD: mechanisms, cellular targets, and therapeutic implications. On the right, cigarette smoke–induced oxidative stress and iron overload drive a self-reinforcing loop of iron accumulation, lipid peroxidation, inflammation, and structural lung damage, leading to emphysema, airway remodeling, and impaired gas exchange. The central panel depicts the core ferroptosis pathway: in multiple lung cell types (airway/alveolar epithelium, endothelium, fibroblasts, macrophages, neutrophils), excess iron and Fenton chemistry increase ROS, promote PUFA lipid peroxidation, deplete glutathione, and cause GPX4 dysfunction, culminating in ferroptotic cell death and DAMPs release. The left panel highlights immune-microenvironment changes, including M1 macrophage polarization, elevated pro-inflammatory cytokines, and structural injury. The bottom box summarizes therapeutic opportunities—iron chelators, System Xc^−^ agonists, and GPX4 activators—to reduce oxidative stress and interrupt the ferroptosis–inflammation feedback cycle in COPD.

Based on the above mechanisms, iron chelators, system Xc^−^ agonists, and GPX4 activity enhancers are considered potential anti-ferroptosis intervention strategies, which have shown feasibility in reducing oxidative stress and structural damage in animal models (122). However, iron is crucial for host immune defense and erythropoiesis, and systemic iron deprivation may pose risks such as anemia and increased susceptibility to infections. Therefore, a more ideal direction is not simply systemic “de-ironization,” but rather targeted regulation in the lung or specific cell lineages (such as airway epithelium vs. alveolar macrophages), combined with traditional antioxidant and anti-inflammatory interventions, to break the self-amplifying loop of ferroptosis–oxidative stress–inflammation–structural remodeling in COPD (123) (**Figure 5**).

### 2.5 Cuproptosis

Cuproptosis is a recently defined form of programmed cell death characterized by the excessive accumulation of free copper ions within cells. These ions directly bind to acetylated TCA cycle proteins (such as DLAT) in the mitochondria, inducing their abnormal aggregation. This aggregation is accompanied by the loss of iron-sulfur cluster proteins, which ultimately triggers mitochondrial protein toxicity stress and energy metabolism collapse (124–126). This process is finely regulated by copper death-related genes such as FDX1, LIPT1, and DLST, creating a high coupling between copper homeostasis, mitochondrial metabolism, and cell fate.

It is noteworthy that existing evidence suggests that cuproptosis does not occur as an “isolated pathway” in COPD, but is more likely embedded in a “PCD network” composed of various forms of programmed cell death, intersecting and cooperating both spatially and temporally with apoptosis, necroptosis, pyroptosis, and ferroptosis. Clinical studies have shown that chronic smokers and COPD patients have elevated copper levels in serum and lung tissues, which are correlated with oxidative stress levels and the degree of lung function impairment (127, 128). Transcriptomic analysis further revealed that various cuproptosis-related genes (such as GLS, DLST, LIPT1) are differentially expressed in COPD lung tissues and are associated with lung function indicators and the frequency of acute exacerbations (129). *In vitro* models treated with CSE or subjected to increased copper load exhibited “cuproptosis-like” phenotypes, such as abnormal mitochondrial structure, TCA cycle dysfunction, and decreased cell viability, occurring concurrently with enhanced oxidative stress, lipid peroxidation, and amplified inflammatory signals, suggesting that cuproptosis may form a metabolic coupling with ferroptosis and mitochondrial stress-type PCD (130, 131). Current evidence indicates that cuproptosis can occur in structural cells such as epithelial cells, endothelial cells, and fibroblasts, and may also affect certain T cell and macrophage populations, with its impact on cytokine profiles and immune metabolic states being more indirectly regulated through mitochondrial function and metabolic reprogramming (132).

From the perspective of immune and inflammatory regulation, the significance of cuproptosis in COPD may be more reflected in “immune metabolic reprogramming” rather than directly inducing immune cell lysis. Multi-omics studies have shown that the expression patterns of cuproptosis-related genes are closely related to the infiltration levels of CD8^+^ T cells, Th1/Th17 cells, and different macrophage subpopulations in lung tissues (133). Proposed mechanisms include that after structural cells undergo cuproptosis, mitochondrial dysfunction and DAMPs release can activate alveolar macrophages and dendritic cells; while the increased mitochondrial copper load in immune cells may reshape their effector functions and inflammatory phenotypes by affecting the OXPHOS-glycolysis balance (134, 135). This process resonates with the recently proposed concept of PANoptosis, where multiple PCD pathways operate in a coordinated manner at common metabolic and inflammatory nodes (such as mitochondrial function, ROS, NLRP3 inflammasome), rather than independently (136).

Furthermore, the imbalance of copper metabolism combined with oxidative stress may enhance the activation threshold of the NLRP3 inflammasome, creating a synergistic amplification effect of cuproptosis with pyroptosis and necroptosis; at the same time, copper-induced mitochondrial stress may also alter the coupling between immune checkpoint signals and metabolic pathways, indirectly affecting T cell exhaustion and the persistent inflammatory state regulated by the PD-1/PD-L1 axis. These findings align with the new paradigm of “immune checkpoint-metabolism-cell death” cross-regulation proposed in PCD immunology research.

**Figure 6:**
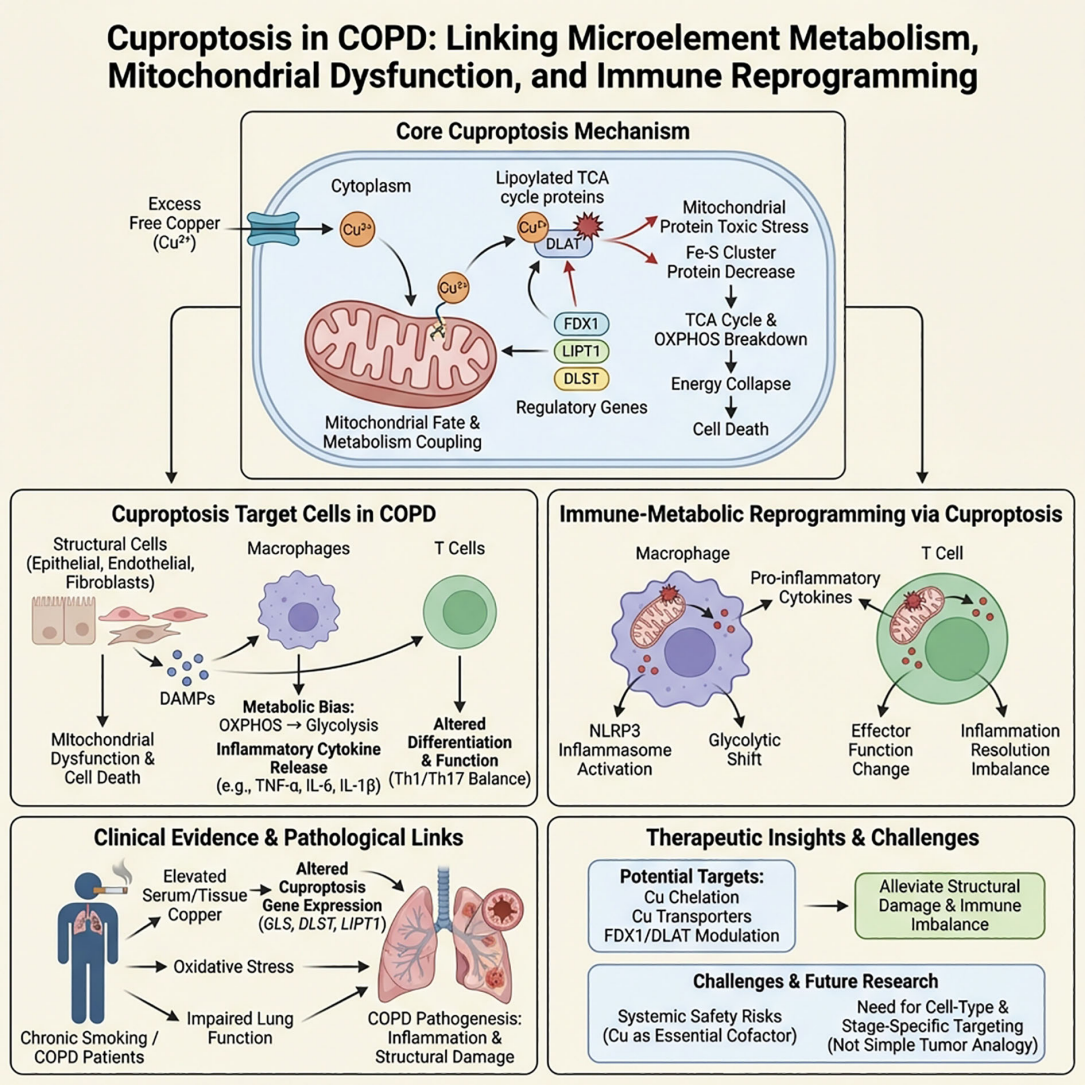
Cuproptosis in COPD: The connection between trace element metabolism, mitochondrial dysfunction, and immune reprogramming. Illustration of the core mechanism: Excess free copper enters cells, binds to acetylated TCA cycle proteins, and aggregates. This process induces a decrease in Fe–S cluster proteins, mitochondrial protein toxicity stress, and energy metabolism collapse, ultimately leading to cell death. The lower left section indicates that bronchial/lung structural cells, macrophages, and T cells are the main target cells, exhibiting mitochondrial damage, DAMPs release, and a metabolic shift from OXPHOS to glycolysis accompanied by upregulation of pro-inflammatory cytokines. The middle section emphasizes that cuproptosis drives NLRP3 inflammasome activation and immune-metabolic reprogramming, leading to changes in effector T cell functions and an imbalance in the resolution of inflammation. The lower right summarizes clinical evidence, such as abnormal free copper and copper transport proteins in the serum/sputum of COPD patients, and proposes potential intervention strategies, including targeting copper transport and key molecules like FDX1/DLAT. It also emphasizes the importance of cautiously assessing systemic copper homeostasis and safety while alleviating structural damage and immune imbalance.

In terms of therapeutic implications, regulating copper homeostasis (such as using copper chelators or modulating copper transporter expression) or directly intervening in cuproptosis-related genes/proteins may theoretically provide new targets for alleviating structural damage and immune imbalance in COPD (137). However, given the essential role of copper in various key enzyme systems, systemic interventions pose significant safety risks, and current evidence mainly comes from bioinformatics analyses and *in vitro* models (138). Future research urgently needs to clarify the specific roles of cuproptosis in different cell types, disease stages, and immune phenotypes in COPD animal models and clinical samples, and evaluate it as part of the PCD network regulatory strategy, rather than simply analogizing conclusions from copper death studies in the tumor field (139) (**Figure 6**).

### 2.6 PCD and the immune microenvironment of COPD

As demonstrated by the various types of PCD described above, the remodeling of the immune microenvironment in COPD is not driven by a single mode of cell death, but rather the result of the superimposed effects of multiple PCDs across different cell types and disease stages: at the level of cell lineage, structural cells (airway/alveolar epithelium, vascular endothelium, smooth muscle, fibroblasts) primarily undergo apoptosis, necroptosis, ferroptosis, and cuproptosis, which together determine barrier integrity, the degree of airway remodeling, and the extent of alveolar destruction; innate immune cells (alveolar macrophages, neutrophils, dendritic cells) dynamically switch between apoptosis, necroptosis, and pyroptosis, and their balance directly affects whether inflammation progresses towards resolution or persistent activation; adaptive immune cells (T/B cells) regulate clonal expansion and exhaustion through apoptosis and metabolism-related modes of cell death, thereby influencing the maintenance of immune memory and the risk of autoimmunity (129). At the level of cytokine/chemokine profiles and tolerance-inflammation balance, efficiently cleared apoptotic cells tend to induce anti-inflammatory factors such as TGF-β and IL-10, promoting immune tolerance and tissue repair (140); while pyroptosis, necroptosis, and some ferroptosis/cuproptosis drive neutrophilic inflammation through IL-1β, IL-18, TNF-α, IL-6, CXCL8, enhancing the Th1/Th17 axis and weakening Treg-mediated immune tolerance. The DAMPs released by different PCD modes (such as HMGB1, oxidized lipids, mtDNA, etc.) amplify tissue damage signals into persistent inflammatory stimuli through PRRs/NLRs (141, 142). In terms of interaction with airway/epithelial integrity, the apoptosis and necroptosis of epithelial and endothelial cells directly determine barrier thickness and permeability, while pyroptosis and ferroptosis particularly significantly disrupt membrane integrity (143); barrier damage not only exposes the basement membrane and ECM, promoting cell adhesion and inflammatory cell infiltration, but also alters local oxygen tension and metabolic microenvironment, which in turn reinforces ROS generation, abnormal iron loading, and inflammasome activation, thus forming a self-amplifying loop of PCD-barrier destruction-immune reprogramming (144, 145). From a translational perspective, different PCD modes represent key “nodes” in the immune microenvironment of COPD: precise regulation targeting these nodes can not only directly affect cell survival and structural damage but also indirectly reshape the cytokine network, immune cell lineage composition, and disease phenotypes (such as frequency of acute exacerbations, degree of airway remodeling, risk of co-infection, etc.) (146). Subsequent sections will further explore the causal chain of “PCD-immune microenvironment-COPD phenotype” in conjunction with **Table 1** and related diagrams, and on this basis, assess the clinical feasibility and potential risks of different targeted strategies.

## 3 The role of PCD in the pathological manifestations of COPD

### 3.1 Mechanisms of cell death in small airway lesions

COPD is a disease characterized by chronic airway inflammation and lung parenchymal damage, with small airway lesions playing a key role in its onset and progression (147). Cell death mechanisms play a crucial role in airway epithelial destruction and fibrosis. Studies have shown that damage to, fibrotic repair of, and death of airway epithelial cells can induce chronic inflammation, ultimately leading to small airway dysfunction (60). In this process, cell death not only disrupts epithelial integrity but also promotes pathological remodeling, thereby creating a vicious cycle. Among the various forms of cell death, necroptosis is particularly prominent in the pathological mechanisms of COPD (148, 149). Necroptosis is a form of cell death with pro-inflammatory characteristics, which exacerbates local inflammatory responses by releasing inflammatory mediators from within the cells (150). In COPD patients, airway epithelial cells trigger necroptosis upon exposure to harmful particulate matter (such as cigarette smoke), a process that not only leads to cell destruction but also stimulates abnormal production of inflammatory mediators, further aggravating pathological changes in the airways (151). Additionally, as COPD progresses, the fibrotic repair of airway epithelial cells continues. Damaged epithelial cells often enter an abnormal repair state due to the persistent presence of inflammation, which may lead to fibrosis (152, 153). Research has shown that specific epithelial cell variants in COPD patients are closely associated with fibrotic lesions, and these variants have the potential to promote fibrosis (154). Abnormal repair of cells not only weakens the barrier function of the airway epithelium but also leads to persistent airway remodeling, worsening the severity of the disease (155). Furthermore, the death of airway epithelial cells is intertwined with the pathogenesis of COPD; cell death is not only a result of disease progression but also an important factor that triggers and exacerbates small airway lesions. As research deepens, it gradually reveals that the mechanisms involved include oxidative stress, inflammatory responses, and cell aging (156). A deeper understanding of these mechanisms may provide new targets for the treatment of COPD, developing more effective intervention strategies to slow disease progression and improve the quality of life for patients.

### 3.2 The process of cell death in the formation of emphysema

Emphysema is one of the main manifestations of COPD, characterized by the destruction of alveolar walls and changes in lung tissue structure, leading to decreased gas exchange efficiency (157). In recent years, studies have shown that PCD plays an important role in the occurrence and development of emphysema. Various forms of cell death, including apoptosis, necroptosis, and autophagic cell death, play their respective roles under different pathological conditions (158). First, smoking is the main risk factor for emphysema, and harmful components in smoke not only cause chronic inflammation but also directly lead to the death of alveolar epithelial cells and alveolar macrophages (159). Studies have found that smoking induces apoptosis and necroptosis, leading to the destruction of alveolar walls and the expansion of air cavities (160). In smoking mouse models, the apoptosis of alveolar epithelial cells significantly increases; this increase is closely related to the deterioration of lung function and changes in lung tissue structure (161, 162). Secondly, research on the mechanisms of cell death shows that iron-regulated ROS plays a key role in the process of programmed cell death (163). Oxidative stress is one of the important mechanisms of pathological changes in COPD: excessive generation of ROS not only directly causes cell damage but also accelerates the destruction of alveolar walls by activating apoptosis and necrosis pathways (164, 165). For example, studies have shown that iron metabolism is closely related to the production of ROS, which promotes the occurrence of emphysema by inducing cell death, and the accumulation of iron is also associated with the dysregulation of autophagy (166). In addition, recent studies on necroptosis have revealed its importance in COPD. Necroptosis can not only trigger inflammatory responses but also exacerbate lung damage by releasing cellular contents (167). In smoking-induced emphysema models, the occurrence of necroptosis is mutually influenced by the release of inflammatory mediators, and this interaction may be one of the important mechanisms for the destruction of alveolar wall structure (25, 148). Overall, PCD mediates the loss of alveolar wall structure through various mechanisms in the formation of emphysema, and a deeper understanding of these mechanisms will help develop new therapeutic strategies to improve the prognosis of COPD patients.

### 3.3 The process of cell death in airflow limitation

COPD is a lung disease characterized by airflow limitation and chronic inflammation. The occurrence of airflow limitation is closely related to various mechanisms of cell death, including apoptosis, necroptosis, and ferroptosis (168). Studies have shown that small airway disease and emphysema are the main pathological features of airflow limitation in COPD, and these structural changes are closely related to the inflammatory responses induced by cell death (169). Among them, necrotic cell death is one of the important pathological mechanisms in COPD. Its occurrence leads to the release of cellular contents, triggering a strong inflammatory response; this response not only damages surrounding healthy cells but also exacerbates airway obstruction (170). Existing studies have found that toxic particulate matter can stimulate epithelial cells to undergo necrotic death, promoting the release of cellular contents and inducing abnormal proliferation of pro-inflammatory mediators; these processes simultaneously weaken the macrophages’ ability to clear dead cells, thereby exacerbating the occurrence of small airway disease and emphysema (171). In addition, ferroptosis also plays an important role in the pathogenesis of COPD. Ferroptosis promotes the accumulation of lipid peroxides, further damaging lung tissue. Common iron metabolism imbalances in patients can exacerbate oxidative stress, trigger cell death and chronic inflammation, thereby driving airway remodeling and worsening airflow limitation (172). It is noteworthy that the relationship between cell death and airflow limitation is reflected not only in the occurrence of inflammation but also manifests as a vicious cycle: the inflammatory response induced by cell death further exacerbates cell damage. For example, inflammatory mediators released by dead cells (such as TNF-α, IL-1β) can accumulate locally, further promoting cell death and the expansion of inflammation, thereby continuously damaging airway structure and exacerbating obstruction (173).

### 3.4 PCD in pulmonary vascular remodeling

Pulmonary vascular remodeling is a key pathological change in the development of COPD and plays a central role in the occurrence and progression of COPD-related pulmonary hypertension (174). Pulmonary endothelial cells not only form a barrier between blood and tissues but also play a critical role in regulating vascular tone, coagulation, and inflammation control (175, 176). In COPD patients, PCD of pulmonary endothelial cells is significantly increased, and pathological stimuli such as long-term anoxia, inflammation, and oxidative stress can lead to abnormal levels of apoptosis and necrosis. The apoptosis and necrosis of endothelial cells increase vascular permeability, causing protein exudation, edema, and inflammation. They also release various cytokines and growth factors, such as PDGF and TGF-β, which stimulate the proliferation and migration of smooth muscle cells. This process leads to vascular wall thickening and lumen narrowing, thereby promoting pulmonary vascular remodeling and the progression of pulmonary hypertension (177, 178). Furthermore, DAMPs-released from necrotic cells, such as HMGB1, can activate signaling pathways in vascular smooth muscle cells, promoting RIPK3 expression and MLKL phosphorylation (179). Oligomerized MLKL translocates to the cell membrane, increasing membrane permeability and inducing necrosis. In addition, RIPK3 can enhance smooth muscle cell migration through pathways such as MAPK and PI3K/Akt, thereby exacerbating pulmonary vascular remodeling and pulmonary hypertension (180).

### 3.5 The remodeling effect of PCD on the microenvironment

**Figure 7:**
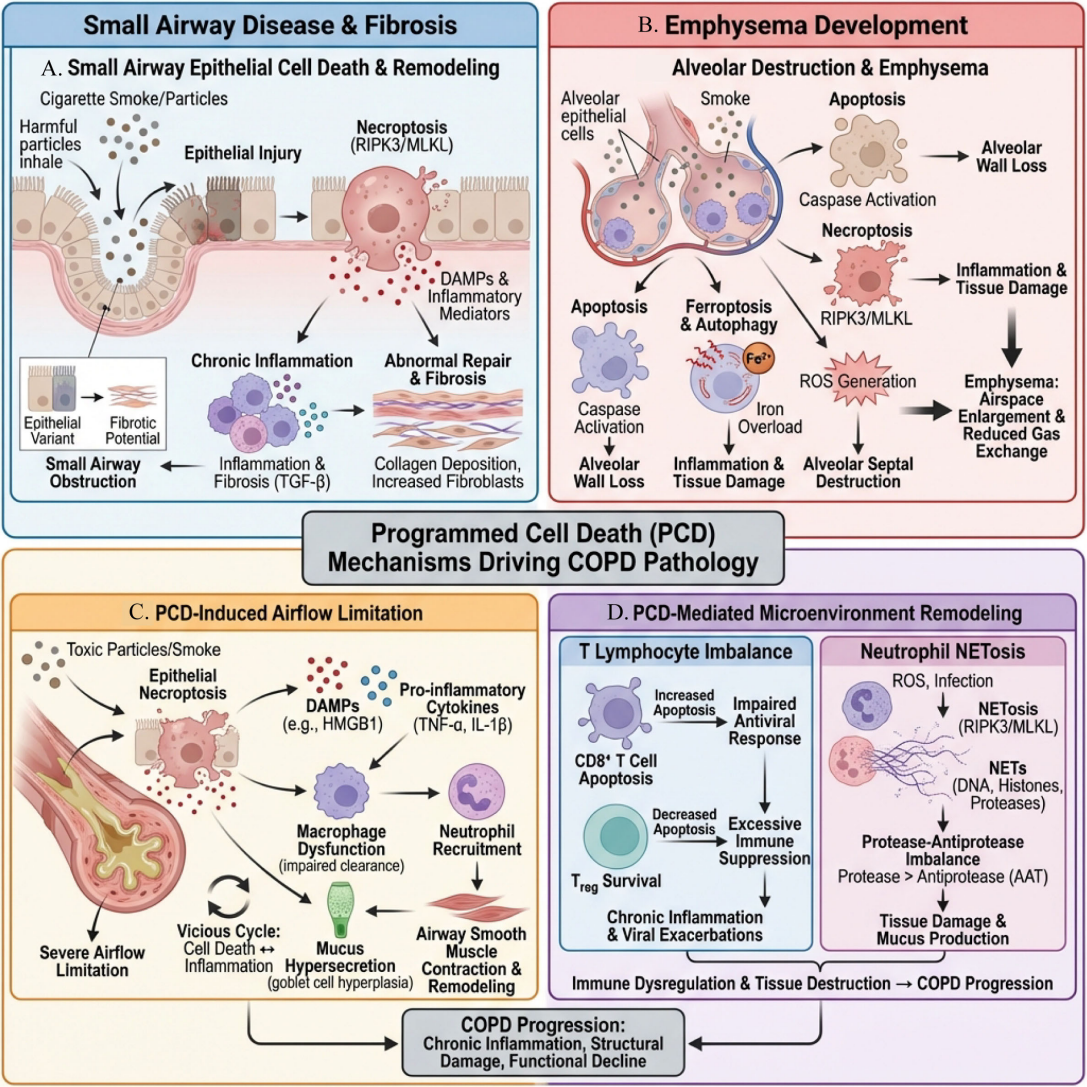
Schematic of the pathological mechanisms of COPD driven by PCD. **(A)** Small airway disease and fibrosis: Cigarette smoke and harmful particles lead to damage of the small airway epithelium and RIPK3/MLKL-mediated necroptosis. This releases DAMPs and inflammatory mediators, resulting in persistent chronic inflammation and abnormal repair, increased collagen deposition, and causing narrowing and obstruction of small airways. **(B)** Formation of emphysema: Alveolar epithelial cells undergo apoptosis, necroptosis, and ferroptosis/autophagy imbalance under smoke and oxidative stress, with ROS and iron overload exacerbating alveolar septal destruction and wall loss, leading to emphysema and impaired gas exchange. **(C)** PCD-induced airflow limitation: Toxic particles trigger epithelial necroptotic cell death, with a large number of DAMPs and pro-inflammatory cytokines recruiting macrophages and neutrophils, forming a vicious cycle of “inflammation–epithelial injury–remodeling,” ultimately resulting in persistent airflow limitation. **(D)** PCD-mediated microenvironment remodeling: Processes including T cell apoptosis and imbalance (exhaustion of CD8^+^ T cells, reduction of Tregs) as well as neutrophil NETosis and inflammation amplification promote immune dysregulation, tissue destruction, and exacerbations. Overall, various modes of PCD synergistically drive chronic inflammation, structural damage, and functional decline in COPD.

In the complex pathological process of COPD, PCD plays an important role in remodeling the pulmonary microenvironment, particularly reflected in the apoptosis of T lymphocytes and immune suppression, as well as the necrosis of neutrophils and the release of proteases (181). First, abnormal apoptosis of T lymphocytes leads to immune imbalance. In COPD patients, the apoptosis of CD8^+^ T cells is significantly increased, while the apoptosis of Treg is decreased (182). CD8^+^ T cells are central to the antiviral response, and the increased apoptosis is closely related to cigarette smoke damage, oxidative stress, and the activation of apoptotic pathways by inflammatory factors such as TNF-α and IL-6. The result is a decline in antiviral function, limited viral clearance, and exacerbated inflammatory responses (183). Clinically, the rate of viral infections and the risk of exacerbation in COPD patients are higher than in healthy populations (184). Conversely, decreased apoptosis of Treg leads to an increased number of these cells, which, although capable of suppressing inflammation to some extent, can excessively suppress immune responses when present in excess. This weakens macrophage and neutrophil functions, allowing inflammation to persist and promoting disease chronicity (185). At the same time, TGF-β released from apoptotic T cells can activate fibroblasts, promoting collagen deposition and leading to pulmonary interstitial fibrosis. Studies have shown that TGF-β expression is elevated in the lung tissues of COPD patients and is correlated with the degree of fibrosis (186, 187). Second, NETosis plays a central role in acute exacerbations. During acute exacerbation, a large number of neutrophils accumulate in the airways and undergo necrosis under the influence of viral or bacterial infections or oxidative stress, releasing extracellular traps (NETs) (188). Studies have found that NETs levels are significantly elevated in COPD patients infected with influenza or Pseudomonas aeruginosa. Excessive ROS can also promote NETosis through the RIPK3/MLKL pathway (110). NETs consist of DNA, histones, and proteases, among which elastase can degrade the elastic fibers of the alveolar wall, triggering emphysema; myeloperoxidase produces strong oxidants, exacerbating tissue damage and inflammation (189–191). Under normal circumstances, antiproteases (such as α1-antitrypsin, AAT) maintain balance and prevent excessive damage. However, in COPD, excessive NETs disrupt this balance, enhancing protease activity while antiproteases are insufficient, leading to destruction of the alveolar wall and excessive mucus secretion, further exacerbating airflow limitation and dyspnea. As the disease progresses, the inflammatory response recruits more neutrophils, releasing more NETs, forming a vicious cycle. Clinical tests indicate that the levels of NETs in the sputum of patients during acute exacerbation are positively correlated with the severity of the condition. By inhibiting excessive NET release or enhancing antiprotease activity, it is expected to alleviate tissue damage and improve patient prognosis (192, 193). In summary, the abnormal cell death pathways of T lymphocytes and neutrophils are important driving forces for immune imbalance, persistent inflammation, and consequent tissue destruction in COPD. The imbalance caused by increased apoptosis of CD8^+^ T cells and decreased apoptosis of Treg leads to a decline in antiviral capability and excessive immune suppression. Meanwhile, neutrophil NETosis disrupts the protease-antiprotease balance through excessive release of NETs, promoting tissue damage and inflammation. These processes interact and jointly shape the microenvironment remodeling in COPD and its disease progression (194) (**Figure 7**).

## 4 The interaction of various forms of PCD in COPD and “death mode selection”

In the chronic inflammation and repeated exacerbation process of COPD, various forms of PCD do not occur in isolation but form a dynamic network characterized by convergence, competition, and mutual transformation around several common “signal hubs” (such as redox imbalance, mitochondrial damage, inflammasome activation, autophagy/mitophagy, metabolic reprogramming, etc.) (195). This network determines whether different cells (airway/alveolar epithelium, alveolar macrophages, neutrophils, T cells, etc.) ultimately undergo apoptosis, ferroptosis, necroptotic apoptosis, or pyroptosis, and profoundly affects whether the immune microenvironment of COPD shifts toward inflammatory resolution or inflammatory amplification (101).

First, autophagy (especially mitophagy) largely acts as a “brake” to inhibit pyroptosis and necroptotic apoptosis (196). In airway epithelial cells and alveolar macrophages exposed to CSE, PINK1-Parkin-mediated mitophagy enhances the clearance of damaged mitochondria, reduces mitochondrial reactive oxygen species (mtROS) and mtDNA leakage, thereby weakening the “second signal” of the NLRP3 inflammasome, inhibiting Caspase-1 activation and GSDMD-mediated pyroptosis (197, 198). At the same time, autophagic lysosomes can directly degrade some components of the inflammasome (such as NLRP3, Pro-IL-1β) and the RIPK1/RIPK3 complex, reducing the tendency for necroptotic apoptosis (199, 200). In contrast, in bronchial biopsies from COPD patients, autophagy markers such as LC3B-II and Beclin-1 are downregulated, autophagic flux is weakened, which is consistent with excessive activation of NLRP3, elevated IL-1β/IL-18, and increased necroinflammatory cell death (201). This suggests that autophagy defects can relieve the negative regulation on pyroptosis and necroptotic apoptosis, making airway/alveolar epithelium and myeloid immune cells more likely to slide from a “reversible stress state” into lytic PCD, promoting neutrophil-dominated inflammatory amplification (202, 203).

Secondly, redox imbalance is a key pivot for the “switching of death modes” between different PCDs, particularly reflected in the competition and transformation between apoptosis and ferroptosis (204). Under moderate oxidative stress, the decrease in mitochondrial membrane potential and the release of cytochrome c more easily trigger classic Caspase-9/3-mediated apoptosis; when iron load significantly increases, lipid peroxidation becomes uncontrolled, and GPX4 function is impaired, the same oxidative environment more readily pushes cells towards the “pathway” of ferroptosis (205). In human bronchial epithelial cells exposed to CSE, both Caspase-3 activation (apoptosis marker) and GPX4 downregulation, along with elevated lipid ROS (ferroptosis marker), can be observed, indicating that under high oxidative stress, the two forms of PCD can occur in parallel or even transform into each other (206). Animal experiments show that the use of iron chelators or GPX4 activators can not only reduce ferroptosis markers but also decrease TUNEL-positive apoptotic cells and the extent of alveolar structural damage (207), indicating that by alleviating iron overload and lipid peroxidation, the “death mode selection” can be redirected from highly lytic, pro-inflammatory ferroptosis back to a relatively “silent” apoptotic phenotype, thereby reducing DAMPs release and subsequent inflammatory amplification (208). Meanwhile, Caspase activity itself is also an important “diversion point”: when TNFR signaling is present and Caspase-8 is inhibited, death signals shift from the apoptotic pathway to RIPK1/RIPK3/MLKL-mediated necroptotic apoptosis, which is particularly prominent in virus-related acute exacerbations and severe oxidative stress contexts (209).

Furthermore, pyroptosis, necroptotic apoptosis, apoptosis, and ferroptosis are not simply parallel but form a higher-level PANoptosis network through common nodes (such as NLRP3, RIPK3, ZBP1, etc.) (210, 211). PANoptosis refers to a “multimodal” cell death program that integrates apoptosis, pyroptosis, and necroptotic apoptosis through the PANoptosome complex, typically involving the coordinated assembly of key molecules such as Caspase-8, RIPK3, ASC, NLRP3, and ZBP1 (212, 213). In models of viral pneumonia and severe infections, alveolar epithelial cells and macrophages can simultaneously exhibit a “PANoptotic” phenotype characterized by the coexistence of apoptosis (cleaved Caspase-3), pyroptosis (GSDMD-N), and necroptotic apoptosis (p-MLKL) markers, accompanied by the release of IL-1β, IL-18, TNF-α, and a large number of DAMPs, triggering an immune pathological response similar to a “cytokine storm.” Although there is currently a lack of direct evidence for PANoptosis in COPD, considering that acute exacerbations of COPD are often triggered by viral/bacterial infections and severe oxidative stress, and that apoptotic, pyroptotic, and necroptotic markers can be observed to overlap in alveolar macrophages and epithelial cells of AECOPD patients, it is reasonable to speculate that within a specific time window of acute exacerbation, PANoptosis is likely to participate in driving airway inflammation uncontrollably and barrier collapse as a “magnified version of lytic death,” becoming a key link connecting infection, inflammatory storm, and structural deterioration (214).

**Figure 8:**
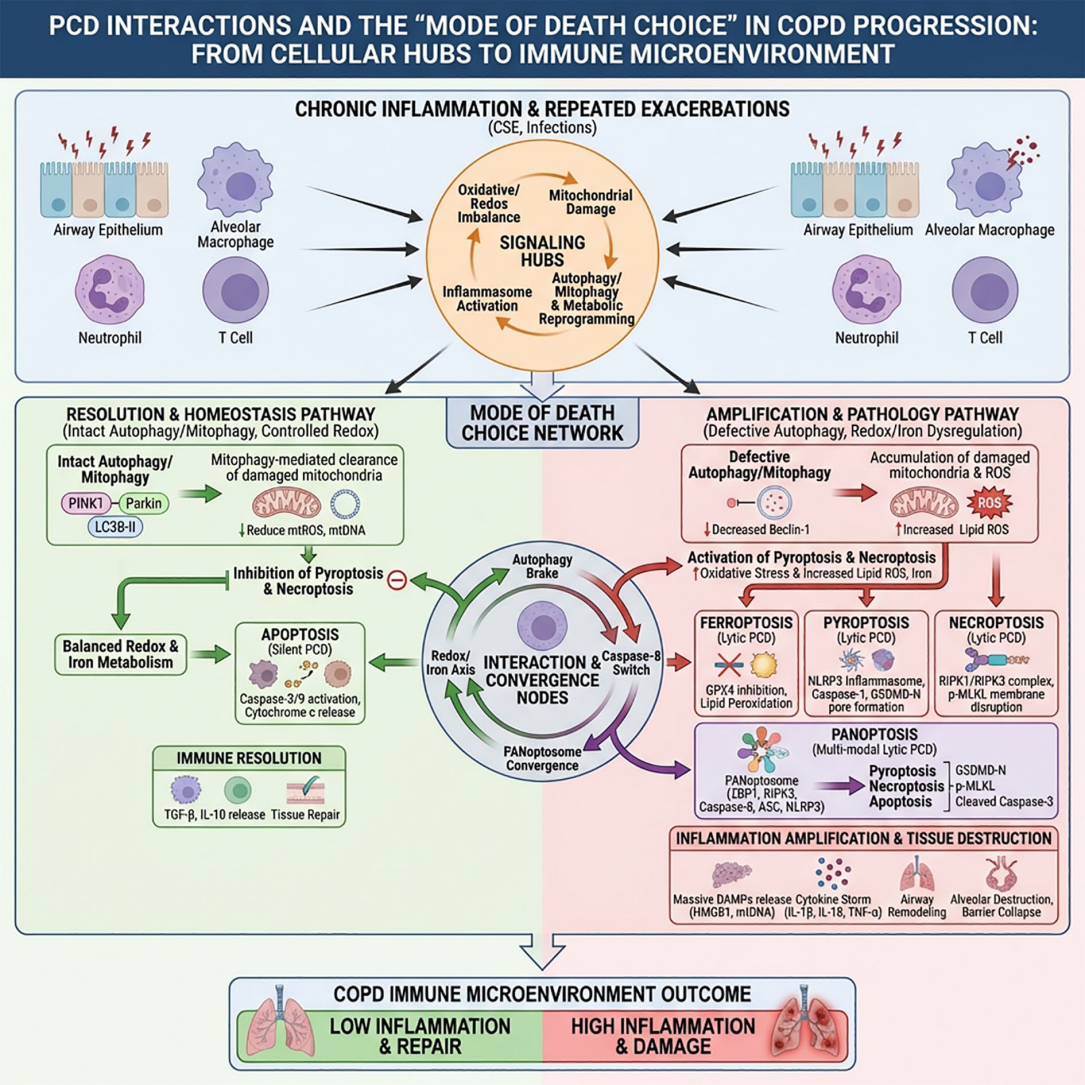
The role of PCD interactions and “mode of death selection” in the progression of COPD. Chronic inflammation and repeated exacerbations (above) expose airway epithelium, alveolar macrophages, and neutrophils to oxidative stress, mitochondrial damage, and inflammasome activation, which act as “signal hubs,” driving autophagy, iron/copper metabolism, and immune reprogramming. The “mode of death selection network” in the middle depicts two pathways: the left green pathway shows that when autophagy/mitophagy is intact and redox is controlled, cells tend toward silent apoptosis and restricted necrosis, inhibiting ferroptosis, pyroptosis, and necroptotic apoptosis, thereby promoting inflammation resolution and tissue repair. The right red pathway indicates that when autophagy is defective and iron and ROS are imbalanced, cells shift toward ferroptosis, pyroptosis, necroptotic apoptosis, and their PANoptosis interactions, releasing large amounts of DAMPs and inflammatory cytokines, thereby amplifying inflammation and tissue damage. Summary below: The “selection and combination” of different PCD pathways determines the outcomes of the COPD immune microenvironment—either a low-inflammation, repairable state or a high-inflammation, persistent damage, and disease progression.

Finally, from the perspective of the overall network, the autophagy-PCD axis, oxidative stress-iron metabolism axis, and PANoptosis jointly determine “which cells die first, what type of death occurs, and which mediators are released” in the COPD immune microenvironment: when autophagy/mitophagy is intact and oxidative stress is controllable, more cells are cleared through relatively silent apoptosis, favoring TGF-β and IL-10 mediated immune resolution; when autophagy is impaired, iron load and ROS levels become uncontrolled, and the inflammasome-RIPK3 node is strongly activated, the death pathway shifts from apoptosis to ferroptosis, necroptotic apoptosis, and pyroptosis, even appearing in the form of PANoptosis, resulting in a “total explosion” of DAMPs/inflammatory factors such as HMGB1, mtDNA, IL-1β/IL-18, pushing the COPD immune microenvironment into a high-inflammatory, high-tissue-destructive malignant stage (27, 215) (**Figure 8**).

This perspective suggests that in the subsequent design of interventions targeting COPD, it is difficult to achieve lasting benefits by targeting a single PCD pathway: enhancing mitophagy, finely regulating redox balance and iron homeostasis, and moderately inhibiting the NLRP3-RIPK3-Caspase axis during the acute exacerbation window may be more conducive to the overall “reprogramming” of the PCD network. This reprogramming shifts it from a PANoptotic, lytic pro-inflammatory death lineage back towards a more controllable apoptotic and inflammatory resolution trajectory, thereby reducing acute exacerbations and structural progression while preserving the host’s anti-infection and immune surveillance capabilities as much as possible.

## 5 The signaling pathways and regulatory mechanisms of PCD

### 5.1 Cross-regulation of intracellular signaling pathways

The cross-regulation of intracellular signaling pathways is crucial in PCD, especially in the pathological mechanisms of COPD. Studies have shown that the mitogen-activated protein kinase (MAPK), nuclear factor kappa-light-chain-enhancer of activated B cells (NF-κB), and p53 pathways have significant roles in the regulation of PCD. These signaling pathways not only regulate cell proliferation and survival but also are crucial in cellular responses to oxidative stress, inflammatory response, and apoptosis (216–218). First, the MAPK signaling pathway plays a central role in regulating cell death and survival. Various factors, including cytokines and growth factors, can trigger activation of this pathway (219). In the context of COPD, pro-inflammatory cytokines and oxidative stress can activate the MAPK pathway, thereby inducing apoptosis or autophagy (220). Research has found that activation of the MAPK pathway is closely related to lung tissue damage and inflammatory response in COPD patients, indicating that this pathway may become a potential target for COPD treatment (221, 222). Secondly, the NF-κB pathway, as a major inflammatory signaling pathway, also plays a critical role in COPD. Activation of NF-κB can trigger the release of various pro-inflammatory cytokines, such as TNF-α and IL-6, which further promote inflammation in the airways and lung tissues (223, 224). In addition, NF-κB is involved in regulating cell survival and death, particularly in response to cellular stress and apoptosis. Studies have shown that excessive activation of the NF-κB pathway may lead to abnormal cell death, thereby exacerbating the pathological process of COPD (225). Finally, p53, as an important tumor suppressor factor, plays a significant role in cellular stress responses. p53 not only functions in DNA damage repair but also limits the proliferation of damaged cells by regulating the cell cycle and guiding apoptosis (226). In COPD, oxidative stress and inflammation can activate p53, thereby inducing apoptosis or other forms of programmed cell death. Research shows that p53 interacts with various non-coding RNAs, which can influence cell fate by regulating the expression and activity of p53 (227).

### 5.2 The impact of metabolic pathways on PCD

Metabolic pathways play a crucial role in PCD, especially in the pathological mechanisms of COPD. Different forms of cell death in PCD are regulated by various intracellular metabolic pathways. First, iron metabolism has a dual role in cell death. Iron is an essential element for various cellular biological processes, including DNA synthesis and energy metabolism (228). However, excess iron promotes ROS generation, leading to oxidative stress and programmed cell death (229). In COPD, environmental factors such as smoking lead to iron accumulation, further promoting oxidative damage and apoptosis within cells (230). Studies have shown that iron metabolism disorders may be closely related to lung tissue damage and cell death in COPD patients, suggesting that regulating iron metabolism may become a new strategy for treating COPD (231). Secondly, lipid metabolism is also crucial in programmed cell death. Lipid peroxidation is one of the key markers of ferroptosis, and this process involves an imbalance in lipid metabolism. Recent studies have shown that changes in lipid metabolism may affect cell survival by regulating the integrity of cell membranes and antioxidant capacity (232). In COPD, abnormalities in lipid metabolism may lead to membrane damage, thereby promoting cell death (233). Therefore, understanding how lipid metabolism interacts with iron metabolism and amino acid metabolism can provide new insights for COPD treatment. Finally, the role of amino acid metabolism in programmed cell death should not be overlooked. Amino acids are not only the building blocks of protein synthesis but also participate in regulating cellular energy metabolism and antioxidant responses. For example, amino acids such as glutamine and cysteine play key roles in maintaining the redox state of cells and synthesizing glutathione (GSH) (234). Glutathione, as an important antioxidant, can scavenge ROS within cells, reducing the risk of cell death. In COPD, dysregulation of amino acid metabolism may lead to decreased antioxidant capacity, thereby exacerbating oxidative damage to cells (235, 236). In summary, the synergistic regulation among iron, lipid, and amino acid metabolism is integral to PCD, especially in the pathological mechanisms of chronic obstructive pulmonary disease. The interactions of these metabolic pathways not only influence cell survival and death processes but also offer potential therapeutic targets for COPD.

### 5.3 The relationship between autophagy and PCD

Autophagy is a process of cellular self-degradation that plays an important role in maintaining cellular homeostasis and clearing damaged cellular components (237). However, recent studies have shown that autophagy plays a dual role in determining cell fate, both protecting cell survival and inducing cell death (238). In COPD research, the relationship between autophagy and PCD is particularly complex, involving various forms of cell death, including apoptosis, autophagic cell death, and necrosis (239). Autophagy typically appears as a protective mechanism in response to intracellular stress and damage. For example, under hypoxic or nutrient-deficient conditions, autophagy maintains cell function and survival by clearing damaged organelles and proteins (240). Research indicates that in the pathological state of COPD, autophagy can help cells resist inflammation and oxidative damage by removing oxidative stress products and damaged mitochondria, thereby delaying cell aging and death (241, 242). However, autophagy may also lead to cell death in certain cases. For instance, during excessive activation of autophagy, cells may undergo autophagic cell death (ACD), which is usually caused by autophagic dysfunction or the accumulation of autophagic structures (243). Studies have shown that in the inflammatory environment of COPD, abnormal activation of autophagy may promote cell apoptosis and autophagic cell death (244). Additionally, there are complex interactions between autophagy and other forms of PCD (such as necrosis and pyroptosis), which further complicate the determination of cell fate (245). In the context of COPD, research shows that the role of autophagy is not only related to cell survival but also closely associated with the regulation of inflammatory responses. Autophagy affects the function of immune cells by regulating intracellular inflammatory signaling pathways, thereby playing an important role in the course of COPD (246, 247). Therefore, understanding the relationship between autophagy and programmed cell death is crucial for developing new treatment strategies for COPD. For example, drug interventions targeting the autophagy pathway may help improve lung function in COPD patients and slow disease progression (248).

### 5.4 Integration of PCD with oxidative stress and inflammatory signaling pathways

**Figure 9:**
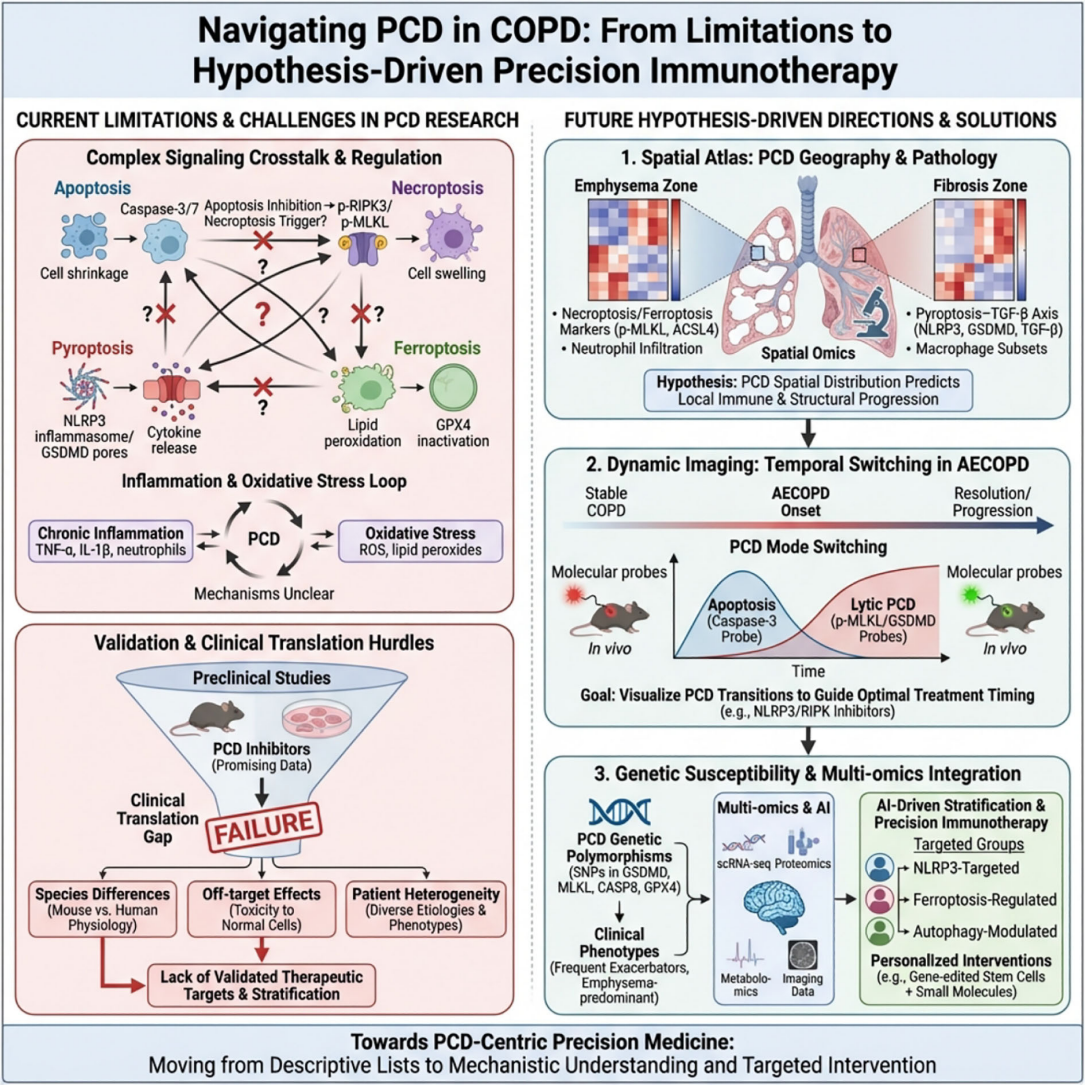
The dilemmas of PCD research in COPD and the hypothesis-driven direction of precise immunotherapy. The left side summarizes current limitations: various forms of programmed cell death (apoptosis, necroptosis, pyroptosis, ferroptosis) have highly overlapping signals, and the inflammation-oxidative stress forms a self-amplifying loop. Significant gaps remain regarding species differences, off-target effects, and patient heterogeneity in translating animal experiments to clinical applications. The right side proposes future paths: ① Construct a “spatial map” of lung tissue, depicting the geographical distribution and pathological associations of PCD in different anatomical regions (such as emphysema and fibrosis areas); ② Develop dynamic imaging and molecular probes to track the transitions between cell death modes in real-time during acute exacerbation-remission processes, providing a basis for optimal intervention timing; ③ Combine genetic susceptibility and multi-omics analysis to identify PCD-related molecular subtypes and drug targets, achieving stratified dosing and personalized treatment. The bottom emphasizes that transitioning to precision medicine centered on “PCD” requires shifting from merely listing pathways to conducting mechanistic analyses and developing targeted intervention strategies.

In the pathological process of COPD, a close interaction exists among cell death, oxidative stress, and inflammatory signaling pathways. Cell death, especially programmed cell death (such as apoptosis, necrosis, and ferroptosis), plays an important role in the progression of COPD (249). Oxidative stress is considered a key factor triggering these forms of cell death, thereby exacerbating pulmonary inflammatory responses. Nrf2 (nuclear factor erythroid 2-related factor 2) is the main transcription factor regulating cellular antioxidant responses, promoting the expression of antioxidant enzymes, thus alleviating oxidative stress damage to cells. In COPD, the activity of Nrf2 is reduced, leading to weakened antioxidant responses, which exacerbates the effects of oxidative stress and promotes cell apoptosis and necrosis (250, 251). Studies have shown that activation of the Nrf2 pathway can alleviate COPD-related inflammatory responses by downregulating the expression of pro-inflammatory factors such as IL-1β, TNF-α, and IL-6 (252). Extracellular signal-regulated kinases 1 and 2 (ERK1/2) are part of the MAPK signaling pathway and play a key role in cell proliferation and survival (253). In COPD, the activation of ERK1/2 is closely related to cell apoptosis and inflammatory responses. Research has found that excessive activation of ERK1/2 can lead to the release of pro-inflammatory cytokines and exacerbate oxidative stress, thereby promoting inflammation and cell death in the lungs (254). Therefore, regulating the activity of ERK1/2 may be a new strategy for controlling the pathological progression of COPD. STAT1 and STAT3 are transcription factors closely related to inflammatory responses. In COPD, STAT1 is usually associated with pro-inflammatory responses, while STAT3 plays a role in regulating cell survival and anti-inflammatory responses (255). Studies have shown that activation of STAT1 can promote the expression of pro-inflammatory cytokines, while activation of STAT3 helps to inhibit inflammation and cell death (256). By regulating these signaling pathways, a balance between cell death and inflammatory responses can be achieved in COPD, thereby reducing the severity of the condition (257). Overall, signaling pathways such as Nrf2, ERK1/2, and STAT1/3 form a complex regulatory network in the cell death, oxidative stress, and inflammatory responses in COPD (**Figure 9**). Abnormal activation or inhibition of these signaling pathways not only affects cell viability but also further aggravates the pathological process of COPD. Therefore, interventions targeting these signaling pathways may provide novel therapeutic approaches for COPD.

## 6 New strategies for COPD treatment provided by PCD

As the pathological roles of various forms of PCD—including apoptosis, necroptosis, pyroptosis, ferroptosis, and cuproptosis—in COPD become clearer, PCD-related pathways are increasingly regarded as potential therapeutic targets. However, most evidence currently remains at the *in vitro* and animal model levels, which makes simple “single-pathway inhibition” difficult to achieve lasting efficacy and may potentially cause unexpected immunological risks due to significant overlaps and “pathway switching” among different PCDs. This section will systematically evaluate the opportunities and limitations of “targeting PCD” through three types of interventions: small molecule inhibitors, immune modulation strategies, and metabolic/redox regulation. It will also specifically discuss the key issues of specificity and timing selection.

### 6.1 Small molecule inhibitors: opportunities and limitations in targeting PCD pathways

In recent years, a number of representative small molecule inhibitors have emerged targeting various forms of necroptosis, pyroptosis, ferroptosis, and cuproptosis, providing conceptual validation for targeted interventions against “pathological PCD” in COPD. However, most of these drugs are still in the *in vitro* experimental or animal model stages, with significant uncertainties regarding pharmacokinetic properties, off-target effects, and COPD-specific safety, necessitating a more cautious and critical evaluation in the review.

In terms of necroptosis, Necrostatin-1 (Nec-1) and its derivatives are the earliest discovered RIPK1 inhibitors, which can block the assembly and activation of the RIPK1–RIPK3–MLKL axis, thereby inhibiting necroptosis (258, 259). In animal models of COPD related to cigarette smoke exposure or viral infection, RIPK1/RIPK3 inhibition can alleviate small airway inflammatory infiltration, alveolar structural damage, and decreased lung compliance, suggesting that necroptosis is a druggable node in the structural damage of COPD (260). However, RIPK1 also participates in NF-κB-mediated cell survival and repair signals, and long-term systemic inhibition may interfere with the body’s adaptive response to infection and tissue damage (261). Additionally, Nec-1 itself has issues such as a short half-life, limited selectivity, and potential off-target kinase inhibition, making it more suitable as an experimental tool rather than a candidate drug for direct clinical use (262). Future efforts should focus on second/third generation highly selective RIPK1/3 inhibitors, combined with COPD-specific pharmacokinetic and safety evaluations, to truly answer whether “necroptosis inhibition has an acceptable risk-benefit ratio.”

In terms of targeting pyroptosis/GSDMD, Disulfiram, a classic alcohol deterrent, has recently been found to covalently modify the key cysteine residue of GSDMD, blocking the insertion of the GSDMD-N terminal into membranes to form pores, thereby inhibiting pyroptosis and the release of IL-1β and IL-18 (263, 264). In models of acute lung injury and infectious inflammation, Disulfiram significantly reduces inflammatory cell infiltration and cytokine storms in lung tissue, providing an attractive “old drug new use” approach for targeting pyroptosis in COPD (265). However, it should be noted that its main indications and dosing regimens are not designed for long-term chronic inflammatory diseases, and there have been reports of adverse reactions such as central nervous system and liver toxicity. Pyroptosis plays an important role in anti-infection defense, and long-term blockade of GSDMD may further weaken the already fragile defense capabilities of COPD patients. At the same time, Disulfiram only cuts off the final link of “pore formation,” which may cause upstream signals of the inflammasome to “detour” to other lytic PCDs (even PANoptosis) (266). Therefore, a more reasonable positioning at present is to regard Disulfiram as a mechanism research tool and an early exploratory intervention, rather than a routine treatment option that can be directly promoted to the COPD population.

In terms of inhibiting ferroptosis, small molecules such as Ferrostatin-1 and Liproxstatin-1 act by clearing lipid peroxyl radicals and stabilizing the membrane structure of PUFA, thereby inhibiting ferroptosis (258). In airway/alveolar epithelial cells treated with CSE and in smoke-exposed mouse models, these drugs can reduce lipid ROS accumulation, partially restore GPX4 function, and alleviate alveolar septal damage and inflammatory infiltration, thereby providing functional evidence supporting the causal role of ferroptosis in COPD structural damage and inflammation amplification (267). However, from a translational perspective, there are still multiple challenges: first, most ferroptosis inhibitors are highly lipophilic, and after oral or injectable administration, their widespread systemic distribution increases potential off-target toxicity; second, ferroptosis is involved not only in COPD but also in anti-tumor and certain pathogen clearance processes, and long-term broad-spectrum inhibition may increase the risks of tumor development and infections; third, there is currently a lack of systematic pharmacokinetic/efficacy and safety data targeting the COPD population (268, 269). Therefore, a more promising approach is to explore localized delivery to the lungs (such as inhalation formulations and lipid nanoparticles) and carrier systems that preferentially target structural cells, enhancing local exposure in the lungs while reducing systemic risks, rather than simply replicating experimental protocols of “systemic administration to inhibit ferroptosis.”

In terms of copper homeostasis and inhibiting “cuproptosis,” copper chelators such as Tetrathiomolybdate have been used to reduce the burden of free copper in diseases like Wilson’s disease, theoretically inhibiting copper death-related protein toxicity stress by alleviating mitochondrial acylated protein-copper aggregation and iron-sulfur cluster protein loss (270). Considering the elevated copper levels and abnormal expression of cuproptosis-related genes associated with immune cell infiltration in COPD patients, regulating copper homeostasis conceptually has certain appeal (271). However, similar to iron, copper is an essential cofactor for various key enzyme systems (such as cytochrome c oxidase, Cu/Zn-SOD), and long-term or excessive chelation may lead to mitochondrial dysfunction, decreased antioxidant capacity, and even hematopoietic and neurological toxicity (272). Currently, there is a lack of any systematic animal and clinical studies with COPD as an indication, so regulating copper homeostasis should be viewed as a highly experimental direction primarily focused on mechanism validation, with a long way to go before it can be considered for clinical strategies in COPD patients (273).

Overall, small molecule inhibitors targeting PCD pathways provide a promising target spectrum for COPD treatment, but at this stage, most remain at the level of “concept validation and mechanism probing” (274). To truly achieve the transition from “PCD mechanisms” to “PCD-targeted therapy,” breakthroughs are still needed in several areas: first, improving target selectivity and optimizing pharmacokinetic properties to reduce systemic off-target effects; second, systematically evaluating the impact of long-term PCD inhibition on immune surveillance and tumor risks, considering the unique susceptibility to infections and comorbidity burden in COPD; third, exploring localized delivery to the lungs and cell-type-specific delivery strategies, limiting interventions to “pathological lytic PCD-rich local areas,” rather than crudely “systemically shutting down cell death” (275). Only by selectively alleviating the most destructive cell death programs while preserving physiological apoptosis and host defenses can PCD-targeted therapy hope to achieve true clinical value in COPD.

### 6.2 Immune modulation strategies: from immune checkpoints to DAMPs/inflammasomes

In COPD, PCD is intricately involved in the local immune microenvironment through the release of DAMPs, remodeling of immune checkpoints, and activation of inflammasomes. Therefore, compared to simply “turning off a certain PCD pathway,” targeting these amplification pathways via immune regulation is more conducive to achieving a balance between “suppressing pathological inflammation” and “preserving host defense” (276).

Firstly, the abnormal PD-1/PD-L1 axis in COPD T cells and antigen-presenting cells suggests that immune checkpoints are involved in the chronicity of the disease: CD4^+^/CD8^+^ T cells exhibit high expression of PD-1 with an “exhausted-like” phenotype, whereas PD-L1 is partially downregulated on dendritic cells, monocytes, and bronchial epithelial cells, which is associated with decreased lung function and enhanced autoimmunity (277, 278). In patients with concomitant NSCLC, PD-1/PD-L1 inhibitors can enhance anti-tumor responses, but existing COPD significantly increases the risk of immune-related adverse events (irAEs) such as pneumonia (279). Therefore, from the perspective of PCD–immune microenvironment, systemic removal of the PD-1/PD-L1 checkpoint should not be regarded as a routine strategy for “treating COPD,” but should be limited to strictly stratified populations with concomitant malignancies and used under close monitoring. A relatively more logical idea is to explore local immune checkpoint “enhancement” rather than systemic “removal”: for example, by using PD-1/PD-L1 agonists, PD-L1-Fc fusion proteins, or small molecules to moderately amplify PD-1/PD-L1 signaling locally in the airways to inhibit overactivated effector T cells. If combined with inhaled formulations or targeted delivery via nanocarriers, it is expected to reshape the local tolerance–inflammation balance in the lungs without significantly weakening systemic anti-infection and anti-tumor capabilities. However, this direction is still mainly at the conceptual and early experimental stage (280).

Secondly, DAMPs and inflammasomes are key nodes in the “translation” of PCD to immune amplification. The transition from apoptosis to necroptosis/panoptosis, as well as membrane rupture during ferroptosis, can lead to massive leakage of HMGB1, mtDNA, and oxidized lipids. These molecules activate macrophages and dendritic cells via TLR4, RAGE, and NLRs, triggering a cytokine storm (281, 282). For example, anti-HMGB1 antibodies, recombinant A box fragments, or HMGB1–RAGE/TLR4 antagonists have shown anti-inflammatory effects and reduced tissue damage in various organ injury models, and are expected to be used in COPD for acute exacerbations or patients with high DAMPs phenotypes, providing short-term intervention in the “PCD–DAMPs–inflammation” feedback loop (283). However, HMGB1 is also involved in tissue repair and anti-infection, and long-term or broad-spectrum blockade may lead to new immune deficiencies. Therefore, it is more suitable to be positioned as an auxiliary strategy for acute phase, short-term use (284).

Inflammasomes, especially the NLRP3 complex, are another immune amplification platform highly coupled with various PCDs: pyroptosis directly relies on the NLRP3–Caspase-1–GSDMD axis, while ROS, mtDNA, and ion flux changes released during ferroptosis and necroptosis can also serve as NLRP3 activation signals (285). The small molecule NLRP3 inhibitor MCC950 significantly reduces IL-1β levels and tissue damage in various inflammation and fibrosis models, and is regarded as an “inhibitor of immune amplification nodes across PCD forms,” theoretically expected to alleviate IL-1-driven neutrophilic inflammation during acute exacerbations in COPD (286). However, the IL-1 axis is also crucial for combating bacterial and some viral infections, especially in elderly COPD populations with multiple comorbidities, where long-term deep suppression may increase the incidence of infections and opportunistic infections. Therefore, it is more suitable to be positioned as a strategy for early acute exacerbations, short-term, and in combination with antimicrobial treatments (287).

Overall, the immune modulation schemes surrounding immune checkpoints, DAMPs, and inflammasomes provide a more refined intervention approach for “PCD–immune microenvironment–COPD” than simple PCD inhibition: On one hand, locally enhancing the PD-1/PD-L1 axis may reshape the tolerance–inflammation balance without significantly increasing the risk of irAEs; On the other hand, selectively weakening the amplification links driven by DAMPs and NLRP3 inflammasomes can achieve “overall noise reduction” in the context of the concurrent involvement of multiple PCD (288). However, these strategies for COPD remain exploratory; to avoid repeating the pitfalls of “broad immunosuppression,” three key issues must be addressed: ① Establish standards for patient stratification based on PCD–immune phenotypes to prevent a “one-size-fits-all” treatment approach; ② Assess long-term outcomes in patients with concomitant tumors, chronic infections, or autoimmune diseases; ③ Optimize drug administration routes and timing, prioritizing local, short-term, and phased interventions in the lungs, keeping risks within acceptable limits.

### 6.3 Metabolism and redox regulation: the upstream “total gate” of PCD interaction

Compared to directly blocking a specific PCD terminal pathway, regulating at the metabolic and redox levels is more in line with the pathological characteristics of multiple PCD interactions amplified in COPD. As previously pointed out, oxidative stress is not only a key driving factor causing apoptosis to shift towards ferroptosis but also a common upstream factor that amplifies NLRP3 inflammasome, necroptosis, and necrotic apoptosis (75). Autophagy/mitophagy, to some extent, inhibits necroptosis, necrotic apoptosis, and ferroptosis. Therefore, the Nrf2–antioxidant axis and mTOR/AMPK–autophagy axis can be regarded as the upstream “total gate” of the PCD network, representing promising therapeutic targets (289).

On one hand, Nrf2 activators (such as dimethyl fumarate, sulforaphane, and other electrophilic small molecules) induce the expression of downstream antioxidant genes like HO-1, NQO1, and GCLC, enhancing GSH synthesis and ROS clearance capacity, thereby weakening lipid peroxidation driven by the Fenton reaction, stabilizing GPX4 and system Xc^−^ function, and potentially inhibiting ferroptosis (290). In smoke exposure or elastase-induced emphysema models, Nrf2 activation can alleviate oxidative stress, inflammatory infiltration, and alveolar structural damage, and some studies suggest that it can downregulate NLRP3 inflammasome activation and IL-1β/IL-18 release, thereby indirectly inhibiting necroptosis (291, 292). However, from a translational perspective, two points must be heeded: ① Long-term, widespread activation of Nrf2 may promote certain tumor cells to gain an “antioxidant advantage,” posing potential tumor-promoting risks in COPD populations with a high background of lung cancer; ② Systematically enhancing antioxidant and detoxification capabilities may alter drug metabolism profiles and inflammatory signal thresholds, affecting the body’s normal response to pathogens and environmental toxins (293). Therefore, Nrf2 activators are more suitable to be positioned as a stage-specific, primarily local pulmonary administration auxiliary strategy in specific phenotypes (significant oxidative stress/iron load, without obvious high-risk tumor factors), rather than a universal solution for all COPD patients.

On the other hand, mTOR and AMPK, as core regulatory factors of classic nutrient/energy sensing pathways and autophagy, have extensive intersections with various forms of PCD. Overactivation of mTOR can inhibit autophagy and mitophagy, exacerbating the accumulation of damaged mitochondria and mtROS, thereby promoting NLRP3 inflammasome activation and necroptosis, necrotic apoptosis; conversely, AMPK activation and mTOR inhibition can enhance autophagic flux, promote the clearance of damaged mitochondria, and to some extent inhibit the inflammasome-necroptosis pathway and buffer ferroptosis (294, 295). In airway epithelial cells treated with CSE and COPD animal models, mTOR inhibitors (such as rapamycin) or AMPK activators (such as metformin, AICAR) have been reported to improve mitochondrial function, reduce ROS levels, alleviate inflammatory infiltration and structural damage, accompanied by upregulation of LC3B-II and enhancement of PINK1/Parkin-mediated mitophagy (296). These results provide experimental support for “indirectly correcting downstream PCD imbalance through metabolic/autophagy axis.” However, this strategy is also not “cost-free”: mTOR inhibitors like rapamycin carry risks of immunosuppression and metabolic disorders, and long-term use may further weaken the anti-infection ability of COPD patients; AMPK activators like metformin, while generally safe, have limited applicability for patients with low body weight, malnutrition, or risk of lactic acidosis (297). Additionally, excessive activation of autophagy may promote cell survival and fibrosis progression in certain contexts, suggesting that “simply amplifying autophagy” does not equate to “inevitable benefits.”

In summary, metabolism and redox regulation are not strategies that traditionally “target a specific PCD endpoint molecule,” but rather reshape the susceptibility and interaction patterns of PCD at the systemic level by regulating the two upstream hubs of Nrf2–antioxidant and mTOR/AMPK–autophagy. Moderately enhancing the antioxidant defense can inhibit the “ignition” of ferroptosis and some lytic PCD; optimizing autophagy/mitophagy can reduce the sources of mtROS and DAMPs, thereby weakening the cascade of necroptosis and necrotic apoptosis. The real challenge lies in identifying metabolic/PCD internal phenotypes that are more likely to benefit from Nrf2 or mTOR/AMPK regulation in the highly heterogeneous COPD population, using oxidative stress biomarkers (such as 8-iso-PGF_2_α, MDA), iron metabolism indicators, mitochondrial function, and autophagic flux-related biological markers, and implementing time-limited interventions within appropriate time windows (such as during acute exacerbation recovery or in high-risk stable populations), rather than long-term, indiscriminately “pulling” these basic metabolic pathways (298, 299). Only under this premise can metabolism and redox regulation truly achieve clinical translational value from pathological mechanisms to “PCD-targeted therapy,” rather than evolving once again into a generalized, non-layered “antioxidant/metabolic modulation” slogan.

### 6.4 Specificity and timing: shifting from “global suppression” to “restoring homeostasis”

Although the pathogenic roles of various PCD mechanisms (necrotic apoptosis, pyroptosis, ferroptosis, cuproptosis, etc.) in COPD are increasingly clear, the corresponding inhibitors and immune/metabolic interventions have also expanded the spectrum of therapeutic targets. However, it must be emphasized that PCD itself is a physiological process that maintains tissue renewal, immune surveillance, and defense against infections (133). Long-term and widespread suppression of cell death without stratification and timing control is likely to damage epithelial renewal and weaken anti-infective and anti-tumor capabilities. Therefore, the true goal of PCD-targeted therapy is to selectively inhibit pathological and lytic PCD (especially necrotic apoptosis, pyroptosis, and ferroptosis) during acute exacerbations and high-inflammatory phenotypes, while preserving homeostatic apoptosis and basal autophagy, thereby “suppressing uncontrolled death and restoring physiological balance,” rather than simply “turning off cell death” (300).

At the level of “specificity,” different PCD-dominant internal phenotypes need to be defined using biomarkers, rather than treating all COPD patients as a homogeneous population. For example: sustained elevation of NLRP3–IL-1β/IL-18, increased GSDMD cleavage and LDH release indicate a “pyroptosis/IL-1 high inflammatory type”; elevated non-heme iron in lung tissue or BALF, increased 4-HNE/MDA and lipid ROS, and downregulation of GPX4 indicate an “iron overload–oxidative stress type”; enhanced phosphorylation of RIPK3/MLKL, elevated HMGB1/ATP commonly seen in infection-triggered acute exacerbations, indicate a “necrotic apoptosis dominant type”; while decreased LC3B-II, accumulation of p62, downregulation of PINK1/Parkin, and increased mtDNA release point to an “autophagy/mitophagy deficiency type” (25, 301). Constructing a “fingerprint” based on PCD–immune–metabolic combination biomarkers is a prerequisite for answering “which type of patients are more likely to benefit from pyroptosis inhibition or ferroptosis inhibition.”

At the level of “timing,” the required direction of PCD regulation differs at different stages for the same patient: during the early phase of acute exacerbation (especially during infection and neutrophilic inflammatory storm), short-term inhibition of the NLRP3–IL-1 axis, necrotic apoptosis, or ferroptosis may be considered to limit irreversible structural damage (302). During the recovery phase of acute exacerbation and early stable phase, greater emphasis should be placed on enhancing autophagy/mitophagy and moderately activating Nrf2 to promote the clearance of damaged mitochondria, reduce oxidative stress, and drive inflammation resolution. In the long-term stable phase, most patients are not suitable for continuous use of potent PCD inhibitors, and PCD-related interventions should only be supplemented to standard treatment in the presence of clearly high-risk internal phenotypes, using low doses, intermittently, and primarily through local lung administration (303).

Therefore, whether PCD-targeted therapy can truly fulfill the promise of “From Pathogenesis to Treatment” does not hinge on how many inhibitors are listed again, but rather on whether it can leverage a feasible biomarker system to selectively “turn down” the most destructive PCD at the appropriate time window and targeting the appropriate internal phenotypes, while preserving homeostatic apoptosis and host defense. This dual design of “specificity + timing” should become the core principle of future related clinical trials and translational research.

## 7 Limitations and future research directions

### 7.1 Current limitations and challenges of PCD in COPD research

In healthy organisms, apoptosis is crucial for maintaining the dynamic balance between the body and tissue cells. A deeper understanding of the molecular mechanisms of COPD may help improve the function of alveolar endothelial cells and the impairment of lung function. Multiple distinct PCD pathways are implicated in COPD, and they engage in complex crosstalk that may amplify tissue injury. Notably, almost all forms of programmed cell death are often co-regulated by various factors and signaling pathways, thereby exacerbating the pathological process of COPD. Although existing preclinical studies have conducted preliminary explorations of PCD and COPD and established a basic understanding, there are still many deficiencies and challenges in mechanistic analysis, therapeutic target validation, and clinical translation.

In terms of mechanism research, although various mechanisms of programmed cell death involved in the pathogenesis of COPD have been confirmed, their interrelationships and regulatory networks still need to be explored in depth. Different types of programmed cell death mechanisms may play their unique roles at different stages of COPD, and there may be phenomena of mutual transformation and cross-regulation. There is a complex relationship between apoptosis and necroptosis; in some cases, the inhibition of apoptosis may trigger the occurrence of necroptosis. However, the current understanding of the specific mechanisms of this mutual transformation and its dynamic changes in COPD is still insufficient. Moreover, the complex interactions between programmed cell death and pathological processes such as inflammatory response and oxidative stress have not been fully elucidated. Inflammatory response and oxidative stress can induce programmed cell death, while programmed cell death may further exacerbate inflammation and oxidative stress, forming a vicious cycle. However, the specific signaling pathways and molecular mechanisms involved still have many unknown areas, which limits a comprehensive understanding of the pathogenesis of COPD.

In terms of therapeutic target validation, although treatment strategies based on programmed cell death provide new research directions for COPD treatment, many potential therapeutic targets are still in the basic research stage, and their effectiveness and safety in preclinical studies have not been fully validated (304). Some apoptosis inhibitors have shown therapeutic effects on COPD in animal experiments, but may face adverse reactions or poor efficacy in human clinical trials. This may be attributed to the differences between animal models and humans, as well as the complexity of human physiological and pathological processes (305). Furthermore, due to the complex etiology of COPD, different causes may lead to different pathological characteristics and molecular mechanisms, making a single therapeutic target potentially unsuitable for all COPD patients. Therefore, how to select therapeutic targets that are highly specific, significantly effective, and safe, and provide personalized treatment for COPD patients with different etiologies, has become an urgent issue to be addressed (306).

Clinical translation is a key step in applying basic research findings to clinical treatment, but it currently faces many obstacles. The transition from basic research to clinical application requires rigorous clinical trial validation, a process that consumes a large amount of time, human resources, and financial resources (307). Many treatment methods that are effective in animal experiments may fail in clinical trials for various reasons. Among them, off-target effects are an important issue. Some programmed cell death inhibitors may affect the physiological functions of other normal cells while inhibiting the death pathways of target cells, leading to adverse reactions. At the same time, the transition from basic cell and rodent model studies to human clinical trials faces species differences; the physiological complexity and immune responses in humans differ from those in animal models, making it difficult to directly extrapolate preclinical research results to humans (308). Finally, current research is mostly limited to cell and preclinical rodent models, lacking large-scale multicenter clinical trials, which hinders the progress of clinical translation (309).

### 7.2 The issues and solutions that need to be addressed in future research

Given the above limitations, future research should move beyond merely listing technical terms such as “multi-omics,” “combinational therapy,” and “AI,” and always focus on the core scientific question of “how PCD shapes COPD immune pathology,” proposing specific hypotheses that can be empirically tested and designing research pathways accordingly. More targeted explorations can be conducted using spatial mapping, dynamic imaging, genetic susceptibility, and multi-omics/AI.

At the spatial mapping level, it is necessary to test the core hypothesis that “PCD geography determines pathological phenotype”: specifically, whether a reproducible “PCD geography” pattern exists in different pathological phenotype regions (emphysema-dominant area and fibrosis-dominant area) within the same COPD lung tissue (310). Specifically, high-throughput partitioned sequencing and labeling can be utilized in the emphysema and fibrosis areas using spatial transcriptomics and spatial proteomics to systematically depict the spatial expression “hot zones” of key pathway molecules (such as CASP3, p-RIPK3/p-MLKL, NLRP3/GSDMD, ACSL4/GPX4, etc.) related to apoptosis, necroptosis, pyroptosis, and ferroptosis. This can be combined with spatial immunohistochemistry/multiplex immunofluorescence to obtain information on neutrophils, different macrophage subpopulations, Th17/Treg ratios, etc. Thereby, it can be determined whether the emphysema area is primarily enriched in necroptosis/ferroptosis signals, whether the fibrosis area is more inclined towards sustained pyroptosis–TGF-β axis activation, and whether this PCD spatial distribution profile can predict local immune infiltration composition and structural progression speed, providing a structural–immune integrated basis for regionally or lesion-type stratified targeted PCD local interventions (311, 312).

At the dynamic imaging level, the process by which death patterns switch over time during acute exacerbations (AECOPD) still lacks *in vivo* readouts, making it necessary to develop molecular imaging probes capable of distinguishing apoptosis, necroptosis, and pyroptosis to test hypotheses such as: the time series changes of the dominant PCD program during AECOPD progression have a quantifiable correspondence with neutrophilic inflammatory load, bacterial/viral load, and clinical outcomes, which can guide the “optimal initiation timing” of NLRP3 inhibitors, RIPK1/3 inhibitors, or Caspase-targeted drugs (313, 314). In terms of technical pathways, fluorescent/radiolabeled tracers targeting activated Caspase-3/9 can be designed to monitor apoptosis, specific probes combining p-MLKL or its downstream membrane conformational changes can be developed to label necroptosis, and probes recognizing GSDMD-N terminal or IL-1β enrichment can reflect pyroptosis hotspots, with longitudinal follow-up in animal models and early clinical studies to depict the time trajectory of switching from apoptosis to lytic PCD, providing a “dynamic visualization of PCD” window for personalized interventions (315).

At the genetic susceptibility level, current clinical internal phenotypes of COPD (such as frequent exacerbator type, emphysema predominant type, chronic bronchitis/mucus hypersecretion type, etc.) still lack direct genetic evidence linked to the PCD axis, making it necessary to systematically detect functional SNPs or copy number variants of key PCD executors (such as GSDMD, MLKL, CASP8, GPX4, etc.) in prospective cohorts, analyzing their associations with AECOPD frequency, CT quantitative emphysema load, small airway wall thickness, IL-1/IL-18 levels, and neutrophil ratios, thereby answering whether these genetic polymorphisms can define different PCD-driven clinical internal phenotypes and predict patient responses to NLRP3 inhibitors, RIPK1/3 inhibitors, or ferroptosis-modulating drugs (316). If validated, this will provide a solid foundation for “PCD genotype-guided immunotherapy” (317).

Based on the above three problem orientations, the role of multi-omics and artificial intelligence should also shift from “trendy concepts” to specific tools serving hypothesis testing and stratification of immune internal phenotypes: at the mechanistic level, by integrating single-cell transcriptomics, single-cell ATAC-seq, proteomics, and metabolomics data, a “PCD–immune metabolism” joint network model can be constructed to predict the programmed cell death tendencies of different cell types under specific stimuli and cross-validate with spatial omics results; at the translational level, machine learning models can be trained using data from large-scale cohorts that include genotypes, PCD-related biomarkers, imaging, and clinical outcomes to achieve unsupervised clustering and risk stratification of “PCD–immune internal phenotypes,” thereby identifying patient subgroups most suitable for NLRP3/ferroptosis/mTOR-autophagy pathway-targeted therapies; in terms of intervention strategies, the design of combination therapy should also revolve around the main line of PCD–immune microenvironment, for example, constructing mesenchymal stem cells or epithelial progenitor cells with “anti-ferroptosis/anti-pyroptosis” capabilities through gene editing or pre-treatment, enabling them to survive in a high oxidative stress lung environment and actively secrete IL-10, TGF-β, or pro-resolving mediators, in combination with small molecule PCD modulators, thus achieving a combined effect of “inhibiting destructive death programs + enhancing reparative immune programs” within the same immune–metabolic framework.

**Figure 10:**
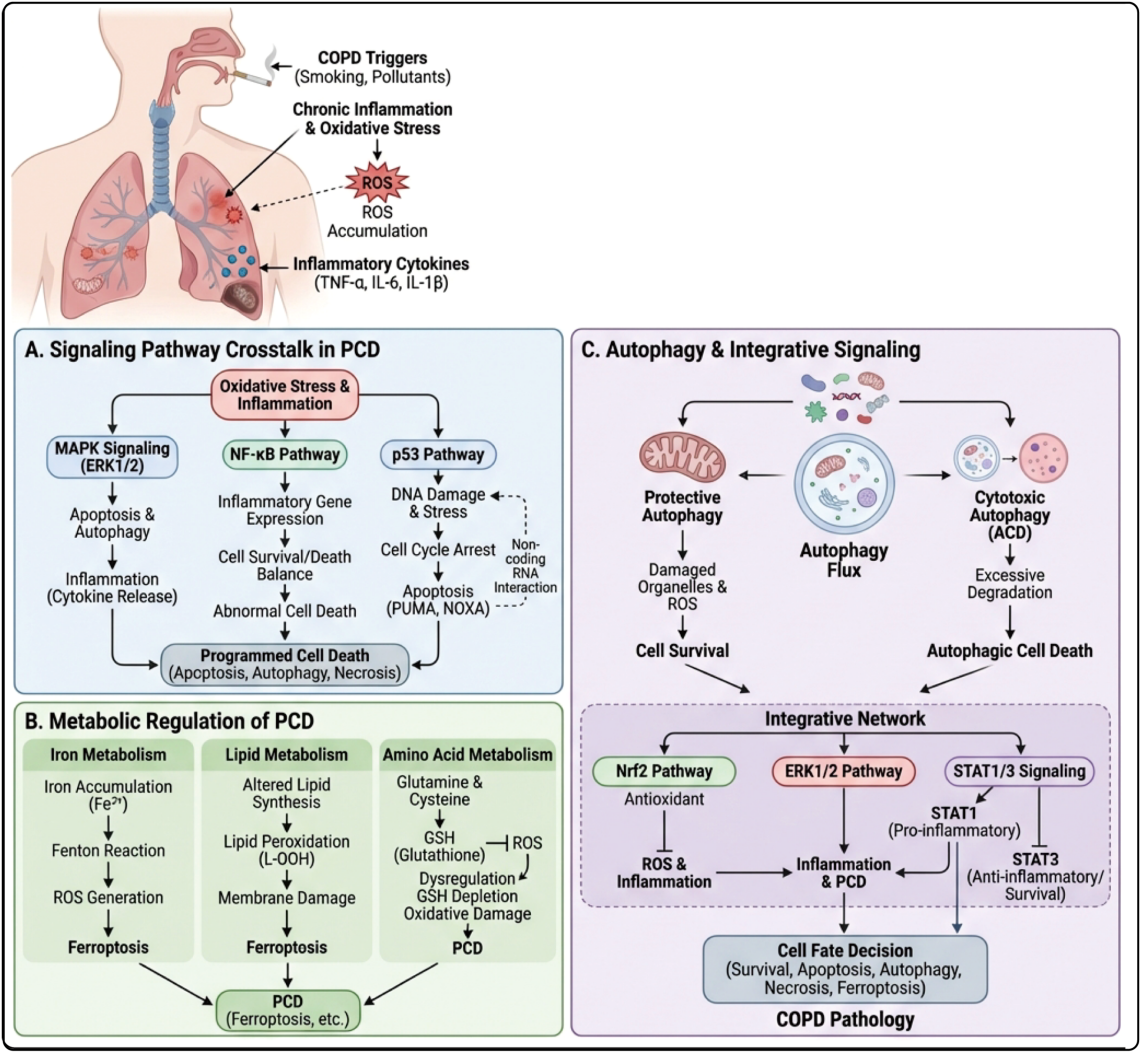
The signaling and metabolic regulation of PCD in COPD. The upper part illustrates that stimuli such as smoking induce chronic inflammation and oxidative stress, leading to the accumulation of ROS and inflammatory factors in the lungs. **(A)** Signal pathway interaction: The MAPK, NF-κB, and p53 pathways jointly regulate the balance of cell survival and death in the context of oxidative stress and inflammation, driving apoptosis, autophagy, necrosis, and other forms of PCD. **(B)** Metabolic regulation: Dysregulation of iron metabolism, lipid peroxidation, and an imbalance in amino acids and glutathione promote metabolic forms of programmed cell death, such as ferroptosis, exacerbating cell damage. **(C)** Autophagy and integrated signaling: Autophagic flux can exert protective effects by clearing damaged organelles, but excessive activation can lead to autophagic cell death, and it intertwines with pathways such as Nrf2, ERK1/2, and STAT1/3, ultimately determining cell fate and thereby participating in chronic inflammation and structural remodeling in COPD.

In summary, future research directions should shift from an abstract technical checklist to clearly defined testable hypotheses centered around PCD–immune pathology: using spatial and single-cell technologies to answer “does PCD geography determine pathological phenotypes,” characterizing “how death patterns dynamically switch during acute exacerbation periods” through *in vivo* imaging, and leveraging genetic epidemiology to analyze “can PCD executor polymorphisms define clinical internal phenotypes.” On this basis, multi-omics and AI can truly become the bridge connecting basic mechanisms, immune internal phenotypes, and clinical trial designs, and targeted interventions based on PCD can move from the conceptual level to operable precise immunotherapy strategies (**Figure 10**).

## 8 Conclusion

This review redefines COPD from a “single death pathway disease” to a chronic inflammation and structural remodeling syndrome driven by “PCD network imbalance.” Apoptosis, necroptosis, pyroptosis, ferroptosis, and cuproptosis are activated in a lineage- and disease stage-specific combination in airway/alveolar epithelium, endothelium, smooth muscle, fibroblasts, and innate/adaptive immune cells. Autophagy/mitophagy intertwines as an upstream “buffer” through the release of DAMPs, the inflammasome–IL-1 axis, PD-1/PD-L1 imbalance, and iron-redox/immunometabolic reprogramming, collectively shaping clinical phenotypes such as neutrophilic inflammation, acute exacerbation susceptibility, and progressive emphysema. Therefore, PCD is not just a terminal event but a central hub connecting tissue damage, immune amplification, and repair failure.

At the immune level, several representative pathways are particularly critical: after the “transition” from apoptosis to necroptosis, pyroptosis, and ferroptosis, HMGB1, mtDNA, and oxidized lipids are released, amplifying focal damage into systemic inflammation via TLRs/RAGE/NLRP3; the NLRP3–Caspase-1–GSDMD axis drives neutrophil aggregation and impairs Treg-mediated tolerance through IL-1β/IL-18–CXCL1/8; iron overload and lipid peroxidation maintain a low but persistent pro-inflammatory state through ferroptosis and macrophage M1 polarization. Therefore, the focus should shift from “whether a certain cell death is important” to “how specific death programs reshape innate and adaptive immune outcomes.”

In terms of potential interventions, the NLRP3 inflammasome–IL-1 axis, iron–lipid peroxidation–GPX4–Nrf2 axis, and mTOR/AMPK–autophagy/mitophagy axis constitute the three key pathways with the most translational prospects. Implementing localized, time-limited, phenotype-stratified interventions around these nodes is more likely to selectively attenuate disruptive signals from the most destructive PCD signals while preserving physiological apoptosis and host defense, guiding the pulmonary immune microenvironment from a neutrophil-dominated high-inflammatory, tissue-destructive state to one characterized by repair, tolerance, and effective immune surveillance.

Future research priorities can be summarized in three points: first, the use of single-cell and spatial omics to construct a “PCD–immune internal phenotype map” of COPD, analyzing the death programs and PANoptosis tendencies of various structural and immune cells at different disease stages; second, developing and validating PCD-related biomarkers and imaging probes such as GSDMD cleavage fragments, p-RIPK3/p-MLKL, 4-HNE/MDA, iron metabolism indicators, and autophagy/mitophagy functional markers for *in vivo* visual monitoring and patient stratification; third, incorporating PCD internal phenotypes into clinical trial designs to test strategies such as NLRP3 inhibitors, ferroptosis modulation, Nrf2 activators, and mTOR/AMPK regulators within appropriate time windows, and systematically evaluating their long-term impacts on acute exacerbations, structural progression, and infection/tumor risks. Overall, the true goal of targeting the PCD pathway is not simply to “inhibit cell death,” but to reprogram the pulmonary immune microenvironment through finely tuned regulation of death programs across different cell types and disease stages, advancing COPD treatment from “blocking damage” to a novel immunotherapy paradigm of “rebuilding homeostasis.”
